# Robust Model Selection Criteria Based on Pseudodistances

**DOI:** 10.3390/e22030304

**Published:** 2020-03-06

**Authors:** Aida Toma, Alex Karagrigoriou, Paschalini Trentou

**Affiliations:** 1 Department of Applied Mathematics, Bucharest University of Economic Studies, 010164 Bucharest, Romania; 2 “Gh. Mihoc - C. Iacob” Institute of Mathematical Statistics and Applied Mathematics, Romanian Academy, 010164 Bucharest, Romania; 3 Department of Statistics and Actuarial-Financial Mathematics, Lab of Statistics and Data Analysis, University of the Aegean, 83200 Karlovasi, Greece; alex.karagrigoriou@aegean.gr (A.K.); sasm17025@sas.aegean.gr (P.T.)

**Keywords:** model selection, minimum pseudodistance estimation, Robustness

## Abstract

In this paper, we introduce a new class of robust model selection criteria. These criteria are defined by estimators of the expected overall discrepancy using pseudodistances and the minimum pseudodistance principle. Theoretical properties of these criteria are proved, namely asymptotic unbiasedness, robustness, consistency, as well as the limit laws. The case of the linear regression models is studied and a specific pseudodistance based criterion is proposed. Monte Carlo simulations and applications for real data are presented in order to exemplify the performance of the new methodology. These examples show that the new selection criterion for regression models is a good competitor of some well known criteria and may have superior performance, especially in the case of small and contaminated samples.

## 1. Introduction

Model selection is fundamental to the practical applications of statistics and there is a substantial literature on this issue. Classical model selection criteria include, among others, the C${}_{p}$-criterion, the Akaike Information Criterion (AIC), based on the Kullback-Leibler divergence, and the Bayesian Information Criterion (BIC) as well as a General Information Criterion (GIC) which corresponds to a general class of criteria which also estimates the Kullback-Leibler divergence. These criteria have been proposed respectively in [1,2,3,4], and represent powerful tools for choosing the best model among different candidate models that can be used to fit a given data set. On the other hand, many classical procedures for model selection are extremely sensitive to outliers and to other departures from the distributional assumptions of the model. Robust versions of classical model selection criteria, which are not strongly affected by outliers, have been proposed for example in [5,6,7]. Some recent proposals for robust model selection are criteria based on divergences and minimum divergence estimators. We recall here, the Divergence Information Criteria (DIC) based on the density power divergences introduced in [8], the Modified Divergence Information Criteria (MDIC) introduced in [9] and the criteria based on minimum dual divergence estimators introduced in [10].

The interest on statistical methods based on divergence measures has grown significantly in recent years. For a wide variety of models, statistical methods based on divergences have high model efficiency and are also robust, representing attractive alternatives to the classical methods. We refer to the monographs [11,12] for an excellent presentation of such methods, for their importance and applications. The pseudodistances that we use in the present paper were originally introduced in [13], where they are called “type-0” divergences, and corresponding minimum divergence estimators have been studied. They are also presented and extensively studied in [14] where they are called γ-divergences, as well as in [15] in the context of decomposable pseudodistances. Like divergences, the pseudodistances are not mathematical metrics in the strict sense of the term. They satisfy two properties, namely the nonnegativity and the fact that the pseudodistance between two probability measures equals to zero if and only if the two measures are equal. The divergences are moreover characterized by the information processing property, that is, the complete invariance with respect to statistically sufficient transformations of the observation space. In general, a pseudodistance may not satisfy this property. We have adopted the term pseudodistance for this reason, but in literature we can also encounter the other terms mentioned above.

The pseudodistances that we consider in this paper have also been used to define robustness and efficiency measures, as well as the corresponding optimal robust M-estimators following the Hampel’s infinitesimal approach in [16]. The minimum pseudodistance estimators for general parametric models have been studied in [15] and consist of minimizing an empirical version of a pseudodistance between the assumed theoretical model and the true model underlying the data. These estimators have the advantage of not requiring any prior smoothing and conciliate robustness with high efficiency, providing a high degree of stability under model misspecification, often with a minimal loss in model efficiency. Such estimators are also defined and studied in the case of the multivariate normal model, as well as for linear regression models in [17,18], where applications for portfolio optimization models are also presented.

In the present paper we propose new criteria for model selection, based on pseudodistances and on minimum pseudodistance estimators. These new criteria have robustness properties, are asymptotically unbiased, consistent and compare well with some other known model selection criteria, even for small samples.

The paper is organized as follows—Section 2 is devoted to minimum pseudodistance estimators and to their asymptotic properties, which will be needed in the next sections. Section 3 presents new estimators of the expected overall discrepancy using pseudodistances, together with corresponding theoretical properties including robustness, consistency and limit laws. The new asymptotically unbiased model selection criteria are presented in Section 3.3, where the case of the univariate normal model and the case of linear regression models are investigated. Applications based on Monte Carlo simulations and on real data, illustrating the performance of the new methodology in the case of linear regression models, are included in Section 4.

## 2. Minimum Pseudodistance Estimators

The construction of new model selection criteria is based on using the following family of pseudodistances (see [15]). For two probability measures *P* and *Q* admitting densities *p* and *q* respectively with respect to the Lebesgue measure, the family of pseudodistances of order γ>0 is defined by

(1) $$R_{\gamma }\left(P,Q\right)=\frac{1}{\gamma +1}\ln\left(\int p^{\gamma }dP\right)+\frac{1}{\gamma (\gamma +1)}\ln\left(\int q^{\gamma }dQ\right)-\frac{1}{\gamma }\ln\left(\int p^{\gamma }dQ\right)$$

and satisfies the limit relation

(2) $$\lim_{\gamma \to 0}R_{\gamma }\left(P,Q\right)=R_{0}\left(P,Q\right),$$

where $R_{0}\left(P,Q\right):=\int \ln\frac{q}{p}dQ$ is the modified Kullback-Leibler divergence.

Let $\left(P_{\theta }\right)$ be a parametric model indexed by θ∈Θ, where Θ is a *d*-dimensional parameter space, and $p_{\theta }$ be the corresponding densities with respect to the Lebesgue measure λ. Let $X_{1},\cdots ,X_{n}$ be a random sample on $P_{\theta_{0}}$, $\theta_{0}\in \Theta$. For γ>0 fixed, a minimum pseudodistance estimator of the unknown parameter $\theta_{0}$ from the law $P_{\theta_{0}}$ is defined by replacing the measure $P_{\theta_{0}}$ in the pseudodistance $R_{\gamma }\left(P_{\theta },P_{\theta_{0}}\right)$ by the empirical measure $P_{n}$ pertaining to the sample, and then minimizing this empirical quantity with respect to θ on the parameter space. Since the middle term in $R_{\gamma }\left(P_{\theta },P_{\theta_{0}}\right)$ does not depend on θ, these estimators are defined by

(3) $${\hat{\theta }}_{n}=\arg\min_{\theta \in \Theta }\left\{\frac{1}{\gamma +1}\ln\left(\int p_{\theta }^{\gamma +1}d\lambda \right)-\frac{1}{\gamma }\ln\left(\frac{1}{n}\sum_{i=1}^{n}p_{\theta }^{\gamma }\left(X_{i}\right)\right),\right\}$$

or equivalently as

(4) $${\hat{\theta }}_{n}=\arg\max_{\theta \in \Theta }\left\{C_{\gamma }{\left(\theta \right)}^{-1}\cdot \frac{1}{n}\sum_{i=1}^{n}p_{\theta }^{\gamma }\left(X_{i}\right)\right\},$$

where $C_{\gamma }\left(\theta \right)={\left(\int p_{\theta }^{\gamma +1}d\lambda \right)}^{\gamma /(\gamma +1)}$. Denoting $h\left(x,\theta \right):=C_{\gamma }{\left(\theta \right)}^{-1}\cdot p_{\theta }^{\gamma }\left(x\right)$, these estimators can be written as

(5) $${\hat{\theta }}_{n}=\arg\max_{\theta \in \Theta }\frac{1}{n}\sum_{i=1}^{n}h\left(X_{i},\theta \right).$$

The optimum given above need not be uniquely defined.

On the other hand,

(6) $$\arg\max_{\theta \in \Theta }\int h\left(x,\theta \right)dP_{\theta_{0}}\left(x\right)=\theta_{0}$$

and here $\theta_{0}$ is the unique optimizer, since $R_{\gamma }\left(P_{\theta },P_{\theta_{0}}\right)=0$ implies $\theta =\theta_{0}$.

Define

$$R_{\gamma }\left(\theta_{0}\right):=\max_{\theta \in \Theta }\int h\left(x,\theta \right)dP_{\theta_{0}}\left(x\right)=\int h\left(x,\theta_{0}\right)dP_{\theta_{0}}\left(x\right).$$

An estimator of $R_{\gamma }\left(\theta_{0}\right)$ is defined by

(7) $${\hat{R}}_{\gamma }\left(\theta_{0}\right):=\max_{\theta \in \Theta }\int h\left(x,\theta \right)dP_{n}\left(x\right)=\max_{\theta \in \Theta }\frac{1}{n}\sum_{i=1}^{n}h\left(X_{i},\theta \right)=\frac{1}{n}\sum_{i=1}^{n}h\left(X_{i},{\hat{\theta }}_{n}\right).$$

The following regularity conditions of the model will be assumed throughout the rest of the paper.

(C1) The density $p_{\theta }\left(x\right)$ has continuous partial derivatives with respect to θ up to the third order (for all *x* λ-a.e.).

(C2) There exists a neighborhood $N_{\theta_{0}}$ of $\theta_{0}$ such that the first-, the second- and the third- order partial derivatives with respect to θ of h(x,θ) are dominated on $N_{\theta_{0}}$ by some $P_{\theta_{0}}$-integrable functions.

(C3) The integrals $\int {\left[\frac{\partial^{2}}{\partial \theta^{2}}h\left(x,\theta \right)\right]}_{\theta =\theta_{0}}dP_{\theta_{0}}\left(x\right)$ and $\int {\left[\frac{\partial }{\partial \theta }h\left(x,\theta \right)\right]}_{\theta =\theta_{0}}{\left[\frac{\partial }{\partial \theta }h\left(x,\theta \right)\right]}_{\theta =\theta_{0}}^{t}dP_{\theta_{0}}\left(x\right)$ exist.

**Theorem** **1.** *Assume that conditions (C1), (C2) and (C3) are fulfilled. Then*

*(a)*  *Let $B:=\left\{\theta \in \Theta ;\;∥\theta -\theta_{0}∥\leq n^{-1/3}\right\}$. Then, as n→∞, with probability one, the function $\theta \mapsto \frac{1}{n}\sum_{i=1}^{n}h\left(X_{i},\theta \right)$ attains a local maximal value at some point ${\hat{\theta }}_{n}$ in the interior of B, which implies that the estimator ${\hat{\theta }}_{n}$ is $n^{1/3}$-consistent.*
*(b)*  *$\sqrt{n}\left({\hat{\theta }}_{n}-\theta_{0}\right)$ converges in distribution to a centered multivariate normal random variable with covariance matrix*
  (8) $$V=S^{-1}MS^{-1},$$
  *where $S:=-\int {\left[\frac{\partial^{2}}{\partial \theta^{2}}h\left(x,\theta \right)\right]}_{\theta =\theta_{0}}dP_{\theta_{0}}\left(x\right)$ and $M:=\int {\left[\frac{\partial }{\partial \theta }h\left(x,\theta \right)\right]}_{\theta =\theta_{0}}{\left[\frac{\partial }{\partial \theta }h\left(x,\theta \right)\right]}_{\theta =\theta_{0}}^{t}dP_{\theta_{0}}\left(x\right)$.*
*(c)*  *$\sqrt{n}\left({\hat{R}}_{\gamma }\left(\theta_{0}\right)-R_{\gamma }\left(\theta_{0}\right)\right)$ converges in distribution to a centered normal variable with variance $\sigma^{2}\left(\theta_{0}\right)=\int h{\left(x,\theta_{0}\right)}^{2}dP_{\theta_{0}}\left(x\right)-{\left(\int h\left(x,\theta_{0}\right)dP_{\theta_{0}}\left(x\right)\right)}^{2}$.*

We refer to [15] for details regarding these estimators and for the proofs of the above asymptotic properties.

## 3. Model Selection Criteria Based on Pseudodistances

Model selection is a method for selecting the best model among candidate models that can be used to fit a given data set. A model selection criterion can be considered as an approximately unbiased estimator of the expected overall discrepancy, a nonnegative quantity which measures the distance between the true unknown model and a fitted approximating model. If the value of the criterion is small, then the approximated candidate model can be chosen. In the following, by applying the same methodology used for AIC, we construct new criteria for model selection using pseudodistances (1) and minimum pseudodistance estimators.

Let $X_{1},\cdots ,X_{n}$ be a random sample from the distribution associated with the true model *Q* with density *q* and let $p_{\theta }$ be the density of a candidate model $P_{\theta }$ from a parametric family $\left(P_{\theta }\right)$, where $\theta \in \Theta \subset R^{d}$.

### 3.1. The Expected Overall Discrepancy

For γ>0 fixed, we consider the quantity

(9) $$W_{\theta }=\frac{1}{\gamma +1}\ln\left(\int p_{\theta }^{\gamma +1}d\lambda \right)-\frac{1}{\gamma }\ln\left(\int p_{\theta }^{\gamma }qd\lambda \right),$$

which is the same as the pseudodistance $R_{\gamma }\left(P_{\theta },Q\right)$ without the middle term that remains constant irrespectively of the model $\left(P_{\theta }\right)$ used.

The target theoretical quantity that will be approximated by an asymptotically unbiased estimator is given by

(10) $$E\left[W_{{\hat{\theta }}_{n}}\right]=E\left[W_{\theta }|\theta ={\hat{\theta }}_{n}\right],$$

where ${\hat{\theta }}_{n}$ is a minimum pseudodistance estimator defined as in (3). The same pseudodistance is used for both $W_{\theta }$ and ${\hat{\theta }}_{n}$. The quantity (10) can be seen as an average distance between *Q* and $\left(P_{\theta }\right)$ up to a constant and is called *the expected overall discrepancy* between *Q* and $\left(P_{\theta }\right)$.

The next Lemma gives the gradient vector and the Hessian matrix of $W_{\theta }$ and is useful for the evaluation of $E[W_{{\hat{\theta }}_{n}}]$ through Taylor expansion.

Throughout this paper, for a scalar function $\varphi_{\theta }\left(\cdot \right)$, the quantity $\frac{\partial }{\partial \theta }\varphi_{\theta }\left(\cdot \right)$ denotes the *d*-dimensional gradient vector of $\varphi_{\theta }\left(\cdot \right)$ with respect to the vector θ and $\frac{\partial^{2}}{\partial \theta^{2}}\varphi_{\theta }\left(\cdot \right)$ denotes the corresponding d×d Hessian matrix. We also use the notations ${\dot{\varphi }}_{\theta }$ and ${\ddot{\varphi }}_{\theta }$ for the first and the second order derivatives of $\varphi_{\theta }$ with respect to θ.

We assume the following conditions allowing derivation under the integral sign:

(C4) There exists a neighborhood $N_{\theta }$ of θ such that

$$\begin{array}{c} \int \underset{t\in N_{\theta }}{\sup}∥\frac{\partial }{\partial t}p_{t}^{\gamma +1}∥d\lambda <\infty ,\;\;\;\int \underset{t\in N_{\theta }}{\sup}∥\frac{\partial }{\partial t}\left[p_{t}^{\gamma }{\dot{p}}_{t}\right]∥d\lambda <\infty . \end{array}$$

(C5) There exists a neighborhood $N_{\theta }$ of θ such that

$$\begin{array}{c} \int \underset{t\in N_{\theta }}{\sup}∥\frac{\partial }{\partial t}p_{t}^{\gamma }∥qd\lambda <\infty ,\;\;\int \underset{t\in N_{\theta }}{\sup}∥\frac{\partial }{\partial t}\left[p_{t}^{\gamma -1}{\dot{p}}_{t}\right]∥qd\lambda <\infty . \end{array}$$

**Lemma** **1.** *Under (C4) and (C5), the gradient vector and the Hessian matrix of $W_{\theta }$ are*

(11) $$\frac{\partial }{\partial \theta }W_{\theta }=\frac{\int p_{\theta }^{\gamma }{\dot{p}}_{\theta }d\lambda }{\int p_{\theta }^{\gamma +1}d\lambda }-\frac{\int p_{\theta }^{\gamma -1}{\dot{p}}_{\theta }qd\lambda }{\int p_{\theta }^{\gamma }qd\lambda }$$

$$\begin{array}{lll} \frac{\partial^{2}}{\partial \theta^{2}}W_{\theta } & = & \frac{\left[\gamma \int p_{\theta }^{\gamma -1}{\dot{p}}_{\theta }{\dot{p}}_{\theta }^{t}d\lambda +\int p_{\theta }^{\gamma }{\ddot{p}}_{\theta }d\lambda \right]\int p_{\theta }^{\gamma +1}d\lambda -\left(\gamma +1\right)\int p_{\theta }^{\gamma }{\dot{p}}_{\theta }d\lambda {\left(\int p_{\theta }^{\gamma }{\dot{p}}_{\theta }d\lambda \right)}^{t}}{{\left(\int p_{\theta }^{\gamma +1}d\lambda \right)}^{2}}\\ & - & \frac{\left[\left(\gamma -1\right)\int p_{\theta }^{\gamma -2}{\dot{p}}_{\theta }{\dot{p}}_{\theta }^{t}qd\lambda +\int p_{\theta }^{\gamma -1}{\ddot{p}}_{\theta }qd\lambda \right]\int p_{\theta }^{\gamma }qd\lambda -\gamma \int p_{\theta }^{\gamma -1}{\dot{p}}_{\theta }qd\lambda {\left(\int p_{\theta }^{\gamma -1}{\dot{p}}_{\theta }qd\lambda \right)}^{t}}{{\left(\int p_{\theta }^{\gamma }qd\lambda \right)}^{2}}. \end{array}$$

When the true model *Q* belongs to the parametric model $\left(P_{\theta }\right)$, hence $Q=P_{\theta_{0}}$ and $q=p_{\theta_{0}}$, the gradient vector and the Hessian matrix of $W_{\theta }$ simplify to

(12) $$\begin{array}{ccc} {\left[\frac{\partial }{\partial \theta }W_{\theta }\right]}_{\theta =\theta_{0}} & = & 0 \end{array}$$

(13) $$\begin{array}{ccc} {\left[\frac{\partial^{2}}{\partial \theta^{2}}W_{\theta }\right]}_{\theta =\theta_{0}} & = & M_{\gamma },\left(\theta_{0}\right) \end{array}$$

where

(14) $$M_{\gamma }\left(\theta_{0}\right):=\frac{\left(\int p_{\theta_{0}}^{\gamma -1}{\dot{p}}_{\theta_{0}}{\dot{p}}_{\theta_{0}}^{t}d\lambda \right)\left(\int p_{\theta_{0}}^{\gamma +1}d\lambda \right)-\left(\int p_{\theta_{0}}^{\gamma }{\dot{p}}_{\theta_{0}}d\lambda \right){\left(\int p_{\theta_{0}}^{\gamma }{\dot{p}}_{\theta_{0}}d\lambda \right)}^{t}}{{\left(\int p_{\theta_{0}}^{\gamma +1}d\lambda \right)}^{2}}.$$

In the following Propositions we suppose that the true model *Q* belongs to the parametric model $\left(P_{\theta }\right)$, hence $Q=P_{\theta_{0}}$, $q=p_{\theta_{0}}$ and $\theta_{0}$ is the value of the parameter corresponding to the true model $Q=P_{\theta_{0}}$. We also say that $\theta_{0}$ is the true value of the parameter (All the proof of the propositions can be seen in the Appendix A).

**Proposition** **1.** *When the true model Q belongs to the parametric model $\left(P_{\theta }\right)$, assuming that (C4) and (C5) are fulfilled for $q=p_{\theta_{0}}$ and $\theta =\theta_{0}$, the expected overall discrepancy is given by*

(15) $$E\left[W_{{\hat{\theta }}_{n}}\right]=W_{\theta_{0}}+\frac{1}{2}E\left[{\left({\hat{\theta }}_{n}-\theta_{0}\right)}^{t}M_{\gamma }\left(\theta_{0}\right)\left({\hat{\theta }}_{n}-\theta_{0}\right)\right]+E\left[R_{n}\right],$$

*where $R_{n}=o(∥{\hat{\theta }}_{n}-\theta_{0}{∥}^{2})$, $M_{\gamma }\left(\theta_{0}\right)$ is given by (14).*

### 3.2. Estimation of the Expected Overall Discrepancy

In this section, we introduce an estimator of the expected overall discrepancy, under the hypothesis that the true model *Q* belongs to the parametric model $\left(P_{\theta }\right)$. Hence, $Q=P_{\theta_{0}}$ and the unknown parameter $\theta_{0}$ will be estimated by a minimum pseudodistance estimator ${\hat{\theta }}_{n}.$

For a given θ∈Θ, a natural estimator of $W_{\theta }$ is defined by

(16) $$Q_{\theta }:=\frac{1}{\gamma +1}\ln\left(\int p_{\theta }^{\gamma +1}d\lambda \right)-\frac{1}{\gamma }\ln\left(\frac{1}{n}\sum_{i=1}^{n}p_{\theta }^{\gamma }\left(X_{i}\right)\right).$$

**Lemma** **2.** *Assuming (C4), the gradient vector and the Hessian matrix of $Q_{\theta }$ are given by*

$$\begin{array}{lll} \frac{\partial }{\partial \theta }Q_{\theta } & = & \frac{\int p_{\theta }^{\gamma }{\dot{p}}_{\theta }d\lambda }{\int p_{\theta }^{\gamma +1}d\lambda }-\frac{\sum_{i=1}^{n}p_{\theta }^{\gamma -1}\left(X_{i}\right){\dot{p}}_{\theta }\left(X_{i}\right)}{\sum_{i=1}^{n}p_{\theta }^{\gamma }\left(X_{i}\right)}\\ \;\frac{\partial^{2}}{\partial \theta^{2}}Q_{\theta } & = & \frac{\left[\gamma \int p_{\theta }^{\gamma -1}{\dot{p}}_{\theta }{\dot{p}}_{\theta }^{t}d\lambda +\int p_{\theta }^{\gamma }{\ddot{p}}_{\theta }d\lambda \right]\int p_{\theta }^{\gamma +1}d\lambda -\left(\gamma +1\right)\int p_{\theta }^{\gamma }{\dot{p}}_{\theta }d\lambda {\left(\int p_{\theta }^{\gamma }{\dot{p}}_{\theta }d\lambda \right)}^{t}}{{\left(\int p_{\theta }^{\gamma +1}d\lambda \right)}^{2}}-\\ & & -\frac{\left[\left(\gamma -1\right)\sum_{i=1}^{n}p_{\theta }^{\gamma -2}\left(X_{i}\right){\dot{p}}_{\theta }\left(X_{i}\right){\dot{p}}_{\theta }{\left(X_{i}\right)}^{t}+\sum_{i=1}^{n}p_{\theta }^{\gamma -1}\left(X_{i}\right){\ddot{p}}_{\theta }\left(X_{i}\right)\right]\sum_{i=1}^{n}p_{\theta }^{\gamma }\left(X_{i}\right)}{{\left(\sum_{i=1}^{n}p_{\theta }^{\gamma }\left(X_{i}\right)\right)}^{2}}\\ & & +\frac{\gamma \left(\sum_{i=1}^{n}p_{\theta }^{\gamma -1}\left(X_{i}\right){\dot{p}}_{\theta }\left(X_{i}\right)\right){\left(\sum_{i=1}^{n}p_{\theta }^{\gamma -1}\left(X_{i}\right){\dot{p}}_{\theta }\left(X_{i}\right)\right)}^{t}}{{\left(\sum_{i=1}^{n}p_{\theta }^{\gamma }\left(X_{i}\right)\right)}^{2}}. \end{array}$$

**Proposition** **2.** *When the true model Q belongs to the parametric model $\left(P_{\theta }\right)$, by imposing the conditions (C1)-(C5), it holds*

(17) $$E\left[Q_{\theta_{0}}\right]=E\left[Q_{{\hat{\theta }}_{n}}\right]+\frac{1}{2}E\left[{\left(\theta_{0}-{\hat{\theta }}_{n}\right)}^{t}M_{\gamma }\left(\theta_{0}\right)\left(\theta_{0}-{\hat{\theta }}_{n}\right)\right]+E\left[R_{n}\right],$$

*where $R_{n}=o(∥{\hat{\theta }}_{n}-\theta_{0}{∥}^{2})$.*

The following result allows to define an asymptotically unbiased estimator of the expected overall discrepancy.

**Proposition** **3.** *When the true model Q belongs to the parametric model $\left(P_{\theta }\right)$, under (C1)-(C5), it holds*

(18) $$\begin{array}{lll} E[W_{{\hat{\theta }}_{n}}] & = & E\left[Q_{{\hat{\theta }}_{n}}\right]+E\left[{\left(\theta_{0}-{\hat{\theta }}_{n}\right)}^{t}M_{\gamma }\left(\theta_{0}\right)\left(\theta_{0}-{\hat{\theta }}_{n}\right)\right]+\\ & & +\frac{1}{2\gamma n}\left[1-\frac{\int p_{\theta_{0}}^{2\gamma +1}d\lambda }{{\left(\int p_{\theta_{0}}^{\gamma +1}d\lambda \right)}^{2}}\right]+E\left[R_{n}\right]+\frac{1}{\gamma }E\left[R_{n}^{\prime }\right], \end{array}$$

*where $R_{n}=o(∥{\hat{\theta }}_{n}-\theta_{0}{∥}^{2})$ and $R_{n}^{\prime }=o(∥\frac{1}{n}\sum_{i=1}^{n}p_{\theta_{0}}^{\gamma }\left(X_{i}\right)-\int p_{\theta_{0}}^{\gamma +1}d\lambda {∥}^{2})$.*

#### 3.2.1. Limit Properties of the Estimator $Q_{{\hat{\theta }}_{n}}$

Under the hypothesis that the true model *Q* belongs to the family of models $\left(P_{\theta }\right)$, hence $Q=P_{\theta_{0}}$, we prove the consistency and the asymptotic normality for the estimator $Q_{{\hat{\theta }}_{n}}$.

Note that

(19) $$\begin{array}{ccc} Q_{{\hat{\theta }}_{n}} & = & \frac{1}{\gamma +1}\ln\left(\int p_{{\hat{\theta }}_{n}}^{\gamma +1}d\lambda \right)-\frac{1}{\gamma }\ln\left(\frac{1}{n}\sum_{i=1}^{n}p_{{\hat{\theta }}_{n}}^{\gamma }\left(X_{i}\right)\right) \end{array}$$

(20) $$\begin{array}{cc} = & -\ln{\left[\frac{\frac{1}{n}\sum_{i=1}^{n}p_{{\hat{\theta }}_{n}}\left(X_{i}\right)}{{\left(\int p_{{\hat{\theta }}_{n}}^{\gamma +1}d\lambda \right)}^{\frac{\gamma }{\gamma +1}}}\right]}^{\frac{1}{\gamma }}=-\ln{\left[{\hat{R}}_{\gamma }\left(\theta_{0}\right)\right]}^{\frac{1}{\gamma }}, \end{array}$$

where $\int p_{{\hat{\theta }}_{n}}^{\gamma +1}d\lambda ={\left[\int p_{\theta }^{\gamma +1}d\lambda \right]}_{\theta ={\hat{\theta }}_{n}}$ and ${\hat{R}}_{\gamma }\left(\theta_{0}\right)$ is given by (7).

First we prove that ${\hat{R}}_{\gamma }\left(\theta_{0}\right)$ is a consistent estimator of $R_{\gamma }\left(\theta_{0}\right)$. Indeed, using Theorem 1 and the fact that $\int \frac{\partial }{\partial \theta }h\left(x,\theta_{0}\right)dP_{\theta_{0}}\left(x\right)=0$, a Taylor expansion of $\frac{1}{n}\sum_{i=1}^{n}h\left(X_{i},\theta \right)$ in ${\hat{\theta }}_{n}$ around $\theta_{0}$ gives

(21) $${\hat{R}}_{\gamma }\left(\theta_{0}\right)=\frac{1}{n}\sum_{i=1}^{n}h\left(X_{i},\theta_{0}\right)+o_{P}\left(n^{-1/2}\right).$$

Using the weak law of large numbers,

(22) $$\frac{1}{n}\sum_{i=1}^{n}h\left(X_{i},\theta_{0}\right)=R_{\gamma }\left(\theta_{0}\right)+o_{P}\left(1\right).$$

Combining (21) and (22), we obtain that ${\hat{R}}_{\gamma }\left(\theta_{0}\right)$ converges to $R_{\gamma }\left(\theta_{0}\right)$ in probability.

Then, using the continuous mapping theorem, since $g\left(t\right)=-\ln t^{\frac{1}{\gamma }}$ is a continuous function, we get

$$Q_{{\hat{\theta }}_{n}}=-\ln{\left[{\hat{R}}_{\gamma }\left(\theta_{0}\right)\right]}^{\frac{1}{\gamma }}\to -\ln{\left[R_{\gamma }\left(\theta_{0}\right)\right]}^{\frac{1}{\gamma }}=W_{\theta_{0}}$$

in probability.

On the other hand, using the asymptotic normality of the estimator ${\hat{R}}_{\gamma }\left(\theta_{0}\right)$ (according to Theorem 1 (c)) together with the univariate delta method, we obtain the asymptotic normality of $Q_{{\hat{\theta }}_{n}}$. The Proposition below summarizes the above asymptotic results.

**Proposition** **4.** *Under (C1)-(C3), when $Q=P_{\theta_{0}}$, it holds*

*(a) $Q_{{\hat{\theta }}_{n}}$ converges to $W_{\theta_{0}}$ in probability.*

*(b) $\sqrt{n}\left(Q_{{\hat{\theta }}_{n}}-W_{\theta_{0}}\right)$ converges in distribution to a centered univariate normal random variable with variance $\frac{\sigma^{2}\left(\theta_{0}\right)}{\gamma^{2}R_{\gamma }{\left(\theta_{0}\right)}^{2}}$, $\sigma^{2}\left(\theta_{0}\right)$ being defined in Theorem 1.*

#### 3.2.2. Robustness Properties of the Estimator $Q_{{\hat{\theta }}_{n}}$

The influence function is a useful tool for describing robustness of an estimator. Recall that, a map *T* defined on a set of probability measures and parameter space valued is a statistical functional corresponding to an estimator ${\hat{\theta }}_{n}$ of the parameter θ, whenever ${\hat{\theta }}_{n}=T\left(P_{n}\right)$, where $P_{n}$ is the empirical measure associated to the sample. The influence function of *T* at $P_{\theta }$ is defined by

$$\mathrm{IF}\left(x;T,P_{\theta }\right):={\left.\frac{\partial T({\tilde{P}}_{\varepsilon x})}{\partial \varepsilon }\right|}_{\varepsilon =0},$$

where ${\tilde{P}}_{\varepsilon x}:=\left(1-\varepsilon \right)P_{\theta }+\varepsilon \delta_{x},$ ε>0, $\delta_{x}$ being the Dirac measure putting all mass at *x*. The gross error sensitivity of the estimator is defined by

$$\gamma^{*}\left(T,P_{\theta }\right)=\underset{x}{\sup}∥\mathrm{IF}\left(x;T,P_{\theta }\right)∥.$$

Whenever the influence function is bounded with respect to *x*, the corresponding estimator is called B-robust (see [19]).

In what follows, for a given γ>0, we derive the influence function of the estimator $Q_{{\hat{\theta }}_{n}}$. The statistical functional associated with this estimator, which we denote by *U*, is defined by

$$U\left(P\right):=\frac{1}{\gamma +1}\ln\left(\int p_{T(P)}^{\gamma +1}d\lambda \right)-\frac{1}{\gamma }\ln\left(\int p_{T(P)}^{\gamma }dP\right),$$

where *T* is the statistical functional corresponding to the used minimum pseudodistance estimator estimator ${\hat{\theta }}_{n}$, namely

$$T\left(P\right):=\arg\underset{\theta }{\sup}C_{\gamma }{\left(\theta \right)}^{-1}\int p_{\theta }^{\gamma }dP$$

where $C_{\gamma }\left(\theta \right)={\left(\int p_{\theta }^{\gamma +1}d\lambda \right)}^{\gamma /(\gamma +1)}$.

Due to the Fisher consistency of the functional *T*, according to (6), we have $T\left(P_{\theta_{0}}\right)=\theta_{0}$ which implies that $U\left(P_{\theta_{0}}\right)=W_{\theta_{0}}$.

**Proposition** **5.** *When $Q=P_{\theta_{0}}$, the influence function of $Q_{{\hat{\theta }}_{n}}$ is given by*

(23) $$\mathrm{IF}\left(x;U,P_{\theta_{0}}\right)=\frac{1}{\gamma }\left[1-\frac{p_{\theta_{0}}^{\gamma }\left(x\right)}{\int p_{\theta_{0}}^{\gamma +1}d\lambda }\right].$$

Note that the influence function of the estimator $Q_{{\hat{\theta }}_{n}}$ does not depend on the estimator ${\hat{\theta }}_{n}$, but depends on the used pseudodistance. Usually, $p_{\theta_{0}}^{\gamma }\left(x\right)$ is bounded with respect to *x* and therefore $Q_{{\hat{\theta }}_{n}}$ is a robust estimator with respect to $W_{\theta_{0}}$.

For comparison at the level of the influence function, we consider the AIC criterion which is defined by

$$AIC=-2\ln(L\left({\hat{\theta }}_{n}\right))+2d,$$

where $L({\hat{\theta }}_{n})$ is the maximum value of the likelihood function for the model, ${\hat{\theta }}_{n}$ the maximum likelihood estimator and *d* the dimension of the parameter. The statistical functional corresponding to the statistic $-2\ln(L\left({\hat{\theta }}_{n}\right))$ is

$$V\left(P\right)=-2\int \ln p_{T(P)}dP$$

where *T* here is the statistical functional corresponding to the maximum likelihood estimator. The influence function of the functional *V* is given by

(24) $$IF\left(x;V,P_{\theta_{0}}\right)=2\left[\int \ln p_{\theta_{0}}dP_{\theta_{0}}-\ln p_{\theta_{0}}\left(x\right)\right].$$

This influence function is not bounded with respect to *x*, therefore the statistic $-2\ln(L\left({\hat{\theta }}_{n}\right))$ is not robust.

**Figure 1:**
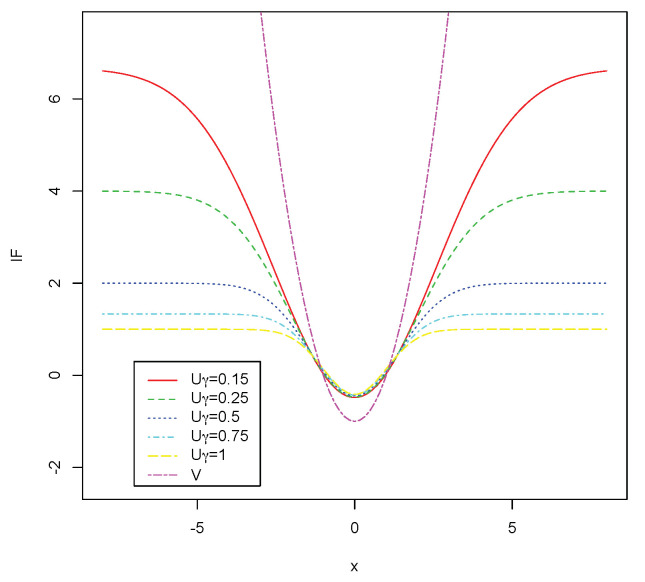
Influence functions in the case of the normal model.

For example, in the case of the univariate normal model, for a positive γ, the influence function (23) writes as

(25) $$\mathrm{IF}\left(x;U,P_{\theta_{0}}\right)=\frac{1}{\gamma }\left(1-\sqrt{\gamma +1}\cdot \exp\left(-\frac{\gamma }{2}{\left(\frac{x-m}{\sigma }\right)}^{2}\right)\right)$$

while the influence function (24) writes as

(26) $$\mathrm{IF}\left(x;V,P_{\theta_{0}}\right)={\left(\frac{x-m}{\sigma }\right)}^{2}-\frac{2m^{2}}{\sigma^{2}}-1$$

(here $\theta_{0}=\left(m,\sigma \right)$). For all the pseudodistances, the influence function (25) is bounded with respect to *x*, therefore the selection criteria based on the statistic $Q_{{\hat{\theta }}_{n}}$ will be robust. On the other hand, the influence function (26) is not bounded with respect to *x*, showing the non robustness of AIC in this case. Moreover, the gross error sensitivities corresponding to these influence functions are $\gamma^{*}\left(U,P_{\theta_{0}}\right)=\frac{1}{\gamma }$ and $\gamma^{*}\left(V,P_{\theta_{0}}\right)=\infty$. These results show that, in the case of the normal model, when γ increases the gross error sensitivity decreases. Therefore, larger values of γ are associated with more robust procedures. For the particular case m=0 and σ=1, the influence functions (25) and (26) are represented in Figure 1.

### 3.3. Model Selection Criteria Using Pseudodistances

#### 3.3.1. The Case of Univariate Normal Family

The criteria that we propose in this section correspond to the case where the candidate model is a univariate normal model from the family of normal models $\left(P_{\theta }\right)$ indexed by θ=(μ,σ). We also suppose that the true model *Q* belongs to $\left(P_{\theta }\right)$.

In the case of the univariate normal model, $M_{\gamma }\left(\theta_{0}\right)$ defined in (14) expresses as

(27) $$M_{\gamma }\left(\theta_{0}\right)=\frac{{\left(\gamma +1\right)}^{2}}{{\left(2\gamma +1\right)}^{3/2}}A\left(\gamma \right)V^{-1},$$

where *V* is the asymptotic covariance matrix given by (8) and the matrix A(γ) is given by

$$A\left(\gamma \right)=\left(\begin{array}{cc} 1 & 0\\ 0 & \frac{3\gamma^{2}+4\gamma +2}{2(2\gamma +1)} \end{array}\right).$$

For small positive values of γ, the matrix A(γ) can be approximated by the identity matrix *I*.

According to Theorem 1, $\sqrt{n}\left({\hat{\theta }}_{n}-\theta_{0}\right)$ is asymptotically multivariate normal and then the statistic $n{\left(\theta_{0}-{\hat{\theta }}_{n}\right)}^{t}V^{-1}\left(\theta_{0}-{\hat{\theta }}_{n}\right)$ has approximately a $\chi_{d}^{2}$ distribution. For large *n*, it holds

(28) $$E\left[{\left(\theta_{0}-{\hat{\theta }}_{n}\right)}^{t}M_{\gamma }\left(\theta_{0}\right)\left(\theta_{0}-{\hat{\theta }}_{n}\right)\right]\approx \frac{{\left(\gamma +1\right)}^{2}}{{\left(2\gamma +1\right)}^{3/2}}\cdot \frac{d}{n}.$$

Also, for the normal model, it holds

(29) $$\frac{\int p_{\theta_{0}}^{2\gamma +1}d\lambda }{{\left(\int p_{\theta_{0}}^{\gamma +1}d\lambda \right)}^{2}}=\frac{\gamma +1}{\sqrt{2\gamma +1}}.$$

Therefore, (18) becomes

(30) $$\begin{array}{c} E\left[W_{{\hat{\theta }}_{n}}\right]≅E\left[Q_{{\hat{\theta }}_{n}}\right]+\frac{{\left(\gamma +1\right)}^{2}}{{\left(2\gamma +1\right)}^{3/2}}\cdot \frac{d}{n}+\frac{1}{2\gamma n}\left[1-\frac{\gamma +1}{\sqrt{2\gamma +1}}\right]+E\left[R_{n}\right]+\frac{1}{\gamma }E\left[R_{n}^{\prime }\right]. \end{array}$$

Using the central limit theorem and asymptotic properties of ${\hat{\theta }}_{n}$ given in Theorem 1, the following hold

(31) $$\begin{array}{ccc} n\cdot o(∥{\hat{\theta }}_{n}-\theta_{0}{∥}^{2}) & = & o_{P}\left(1\right), \end{array}$$

(32) $$\begin{array}{ccc} n\cdot o(∥\frac{1}{n}\sum_{i=1}^{n}p_{\theta_{0}}^{\gamma }\left(X_{i}\right)-\int p_{\theta_{0}}^{\gamma +1}d\lambda {∥}^{2}) & = & o_{P}\left(1\right). \end{array}$$

Using (30), (31) and (32) we obtain:

**Proposition** **6.** *For the univariate normal family, an asymptotically unbiased estimator of the expected overall discrepancy is given by*

(33) $$Q_{{\hat{\theta }}_{n}}+\frac{{\left(\gamma +1\right)}^{2}}{{\left(2\gamma +1\right)}^{3/2}}\cdot \frac{d}{n}+\frac{1}{2\gamma n}\left[1-\frac{\gamma +1}{\sqrt{2\gamma +1}}\right],$$

*where ${\hat{\theta }}_{n}$ is a minimum pseudodistance estimator given by (3).*

Under the hypothesis that $\left(P_{\theta }\right)$ is the univariate normal model, as we supposed in this subsection, the function *h* writes as

(34) $$h\left(x,\theta \right)={\left(\sqrt{\gamma +1}\right)}^{\gamma /(\gamma +1)}\cdot {\left(\sigma \sqrt{2\pi }\right)}^{-\gamma /(\gamma +1)}\cdot \exp\left(-\frac{\gamma }{2}{\left(\frac{x-m}{\sigma }\right)}^{2}\right)$$

and it can be easily checked that all the conditions (C1)–(C5) are fulfilled. Therefore we can use all results presented in the preceding subsections, such that Proposition 6 is fully justified.

Moreover, the selection criteria based on (33) are consistent on the basis of Proposition 4. It should also be noted that the bias correction term in (33) decreases slowly as the parameter γ increases staying always very close to zero $\left(∼10^{-2}\right)$. As expected, the larger the sample size the smaller the bias correction. As we saw in Section 3.2.2, since the gross error sensitivity of $Q_{{\hat{\theta }}_{n}}$ is $\gamma^{*}\left(U,P_{\theta_{0}}\right)=\frac{1}{\gamma }$, larger values of γ are associated with more robust procedures. On the other hand, the approximation of A(γ) with the identity matrix is realized for values of γ close to zero. Thus, positive values of γ smaller than 0.5 for example could represent choices satisfying the robustness requirement and the approximation of A(γ) through the identity matrix, approximation which is necessary to construct the criterion in this case.

#### 3.3.2. The Case of Linear Regression Models

In the following, we adapt the pseudodistance based model selection criterion in the case of linear regression models. Consider the linear regression model

(35) $$Y=\alpha +\beta^{t}X+e$$

where e∼N(0,σ) and *e* is independent of *X*. Suppose we have a sample given by the i.i.d. random vectors $Z_{i}=\left(X_{i},Y_{i}\right)$, i=1,…,n, such that $Y_{i}=\alpha +\beta^{t}X_{i}+e_{i}$.

We consider the joint distribution of the entire data and write a pseudodistance between the theoretical model and the true model corresponding to the data. Let $P_{\theta }$, θ:=(α,β,σ), be the probability measure associated to the theoretical model given by the random vector Z=(X,Y) and *Q* the probability measure associated to the true model corresponding to the data. Denote by $p_{\theta }$, respectively by *q* the corresponding densities. For γ>0, the pseudodistance between $P_{\theta }$ and *Q* is defined by

(36) $$\begin{array}{lll} R_{\gamma }\left(P_{\theta },Q\right) & := & \frac{1}{\gamma +1}\ln\left(\int p_{\theta }^{\gamma }\left(x,y\right)dP_{\theta }\left(x,y\right)\right)+\frac{1}{\gamma (\gamma +1)}\ln\left(\int q^{\gamma }\left(x,y\right)dQ\left(x,y\right)\right)-\\ & & -\frac{1}{\gamma }\ln\left(\int p_{\theta }^{\gamma }\left(x,y\right)dQ\left(x,y\right)\right). \end{array}$$

Similar to [18], since the middle term above does not depend on $P_{\theta }$, a minimum pseudodistance estimator of the parameter $\theta_{0}=\left(\alpha_{0},\beta_{0},\sigma_{0}\right)$ is defined by

(37) $${\hat{\theta }}_{n}=\left({\hat{\alpha }}_{n},{\hat{\beta }}_{n},{\hat{\sigma }}_{n}\right)=\arg\min_{\alpha ,\beta ,\sigma }\left\{\frac{1}{\gamma +1}\ln\left(\int p_{\theta }^{\gamma }\left(x,y\right)dP_{\theta }\left(x,y\right)\right)-\frac{1}{\gamma }\ln\left(\int p_{\theta }^{\gamma }\left(x,y\right)dP_{n}\left(x,y\right)\right)\right\},$$

where $P_{n}$ is the empirical measure associated with the sample. This estimator can be written as

(38) $${\hat{\theta }}_{n}=\left({\hat{\alpha }}_{n},{\hat{\beta }}_{n},{\hat{\sigma }}_{n}\right)=\arg\min_{\alpha ,\beta ,\sigma }\left\{\frac{1}{\gamma +1}\ln\left(\int \phi_{\sigma }^{\gamma +1}\left(e\right)de\right)-\frac{1}{\gamma }\ln\left(\frac{1}{n}\sum_{i=1}^{n}\phi_{\sigma }^{\gamma }\left(Y_{i}-\alpha -\beta^{t}X_{i}\right)\right)\right\},$$

where $\phi_{\sigma }$ is the density of the random variable e∼N(0,σ). Then, the estimator $Q_{{\hat{\theta }}_{n}}$ can be written as

(39) $$Q_{{\hat{\theta }}_{n}}=\min_{\alpha ,\beta ,\sigma }\left\{\frac{1}{\gamma +1}\ln\left(\frac{1}{{\left(\sigma \sqrt{2\pi }\right)}^{\gamma }\sqrt{\gamma +1}}\right)-\frac{1}{\gamma }\ln\left(\frac{1}{n}\sum_{i=1}^{n}\frac{1}{{\left(\sigma \sqrt{2\pi }\right)}^{\gamma }}\cdot \exp\left(-\frac{\gamma }{2\sigma^{2}}{\left(Y_{i}-\alpha -\beta^{t}X_{i}\right)}^{2}\right)\right)\right\}.$$

In order to construct an asymptotic unbiased estimator of the expected overall discrepancy in the case of the linear regression models, we evaluated the second and the third terms from (18).

For values of γ close to 0 (γ smaller than 0.3), we found the following approximation of the matrix $M_{\gamma }\left(\theta_{0}\right)$

(40) $$M_{\gamma }\left(\theta_{0}\right)≃\frac{{\left(\gamma +1\right)}^{2}}{{\left(2\gamma +1\right)}^{3/2}}V^{-1}\left(\begin{array}{cc} I & 0\\ 0 & \frac{3\gamma^{2}+4\gamma +2}{2\gamma +1}, \end{array}\right)$$

where *V* is the asymptotic covariance matrix of ${\hat{\theta }}_{n}$ and *I* is the identity matrix. We refer to [15] for the asymptotic properties of the minimum pseudodistance estimators in the case of linear regression models. Since $\sqrt{n}\left(\hat{\theta_{n}}-\theta_{0}\right)$ is asymptotically multivariate normal distributed, using the $\chi^{2}$ distribution, we obtain the approximation

(41) $$E\left[{\left(\hat{\theta_{n}}-\theta_{0}\right)}^{t}M_{\gamma }\left(\theta_{0}\right)\left(\hat{\theta_{n}}-\theta_{0}\right)\right]≃\frac{1}{n}\cdot \frac{{\left(\gamma +1\right)}^{2}}{{\left(2\gamma +1\right)}^{3/2}}\left[\left(d-1\right)+\frac{3\gamma^{2}+4\gamma +2}{2(\gamma +1)(2\gamma +1)}\right].$$

Also, the third term in (18) is given by

(42) $$\frac{1}{2\gamma n}\left[1-{\left(\frac{\gamma +1}{\sqrt{2\gamma +1}}\right)}^{d}\right].$$

Then, according to Proposition 3, an asymptotically unbiased estimator of the expected overall discrepancy is given by

(43) $$Q_{{\hat{\theta }}_{n}}+\frac{1}{n}\cdot \frac{{\left(\gamma +1\right)}^{2}}{{\left(2\gamma +1\right)}^{3/2}}\left[\left(d-1\right)+\frac{3\gamma^{2}+4\gamma +2}{2(\gamma +1)(2\gamma +1)}\right]+\frac{1}{2\gamma n}\left[1-{\left(\frac{\gamma +1}{\sqrt{2\gamma +1}}\right)}^{d}\right],$$

where $Q_{{\hat{\theta }}_{n}}$ is given by (39). Note that, using the asymptotic properties of ${\hat{\theta }}_{n}$ and the central limit theorem, the last two terms in (18) of Proposition 3 are $o_{P}\left(\frac{1}{n}\right)$.

When we compare different linear regression models, as in Section 4 below, we can ignore the terms depending only on *n* and γ in (43). Therefore, we can use as model selection criterion the simplified expression

(44) $$Q_{{\hat{\theta }}_{n}}+\frac{{\left(\gamma +1\right)}^{2}}{{\left(\sqrt{2\gamma +1}\right)}^{3}}\cdot \frac{d}{n}-\frac{1}{2\gamma n}{\left(\frac{\gamma +1}{\sqrt{2\gamma +1}}\right)}^{d},$$

which we call Pseudodistance based Information Criterion (PIC).

## 4. Applications

### 4.1. Simulation Study

In order to illustrate the performance of the PIC criterion (44) in the case of linear regression models, we performed a simulation study using for comparison the model selection criteria AIC, BIC and MDIC. These criteria are defined respectively by

$$AIC=n\log{\hat{\sigma }}_{p}^{2}+2\left(p+2\right)$$

$$BIC=n\log{\hat{\sigma_{p}}}^{2}+\left(p+2\right)\log n,$$

where *n* the sample size, *p* the number of covariates of the model and ${\hat{\sigma }}_{p}^{2}$ the classical unbiased estimator of the variance of the model,

$$MDIC=nMQ_{\hat{\theta }}+{\left(2\pi \right)}^{-\alpha /2}{\left(1+\alpha \right)}^{2+p/2}p$$

with α=0.25 and

$$MQ_{\hat{\theta }}=-\left[\left(1+\alpha^{-1}\right)\frac{1}{n}\sum_{n=1}^{n}f_{\hat{\theta }}^{\alpha }\left(X_{i}\right)\right],$$

where $\hat{\theta }$ is a consistent estimate of the vector of unknown parameters involved in the model with *p* covariates and $f_{\hat{\theta }}$ is the associated probability density function. Note that MDIC is based on the well known BHHJ family of divergence measures indexed by a parameter α>0 and on the minimum divergence estimating method for robust parameter estimation (see [20]). The value of α=0.25 was found in [9] to be an ideal one for a great variety of settings. The above three criteria have been chosen to be used in this comparative study with PIC not only due to their popularity, but also due to their special characteristics. Indeed, AIC is the classical representative of asymptotically efficient criteria, BIC is known to be consistent, while MDIC is associated with robust estimations (see e.g., [20,21,22,23]).

Let $X_{1},X_{2},X_{3},X_{4}$ be four variables following respectively the normal distributions N(0,3), N(1,3), N(2,3) and N(3,3). We consider the model

$$Y=a_{0}+a_{1}X_{1}+a_{2}X_{2}+\varepsilon$$

with $a_{0}=a_{1}=a_{2}=1$ and ε∼N(0,1). This is the uncontaminated model. In order to evaluate the robustness of the new PIC criterion, we also consider the contaminated model

$$Y=d_{1}\left(a_{0}+a_{1}X_{1}+a_{2}X_{2}+\varepsilon \right)+d_{2}\left(a_{0}+a_{1}X_{1}+a_{2}X_{2}+\varepsilon^{*}\right)$$

where $\varepsilon^{*}∼N\left(5,1\right)$ and $d_{1},d_{2}\in \left[0,1\right]$ such that $d_{1}+d_{2}=1$. Note that for $d_{1}=1$ and $d_{2}=0$ the uncontaminated model is obtained.

The simulated data corresponding to the contaminated model are

$$Y_{i}=d_{1}\left(1+X_{1,i}+X_{2,i}+\varepsilon_{i}\right)+d_{2}\left(1+X_{1,i}+X_{2,i}+\varepsilon_{i}^{*}\right),$$

for i=1,⋯,n, where $X_{1,i},X_{2,i},\varepsilon_{i},\varepsilon_{i}^{*}$ are values of the variables $X_{1},X_{2},\varepsilon ,\varepsilon^{*}$ independently generated from the normal distributions N(1,3), N(2,3), N(0,1), N(5,1) correspondingly.

With a set of four possible regressors there are $2^{4}-1=15$ possible model specifications that include at least one regressor. These 15 possible models constitute the set of candidate models in our study. More precisely, this set contains the full model ($X_{1},X_{2},X_{3},X_{4}$) given by

$$Y=b_{0}+b_{1}X_{1}+b_{2}X_{2}+b_{3}X_{3}+b_{4}X_{4}+\varepsilon$$

as well as all 14 possible subsets of the full model consisting of one ($X_{j_{1}})$, two ($X_{j_{1}},X_{j_{2}}$) and three ($X_{j_{1}},X_{j_{2}},X_{j_{3}}$) of the four regressors $X_{1},X_{2},X_{3}$ and $X_{4}$, with $j_{1}\neq j_{2}\neq j_{3}$, $j_{i}\in \left\{1,2,3,4\right\}$ and i=1,2,3.

In our simulation study, for several values of the parameter γ associated with the pseudodistance, we compared the new criterion PIC with the other model selection criteria. Different levels of contamination and different sample sizes have been considered. In the examples presented in this work, $d_{1}\in \left\{0.8,0.9,0.95,1\right\}$ and n∈{20,50,100}. Additional examples for n=30,75,200,500 have been analyzed (results not shown) with similar findings (see below). For each setting, fifty experiments were performed in order to select the best model among the available candidate models. In the framework of each of the fifty experiments, on the basis of the simulated observations, the value of each of the above model selection criteria was calculated for each of the 15 possible models. Then, for each criterion, the 15 candidate models were ranked from 1st to 15th according to the value of the criterion. The model chosen by a given criterion is the one for which the value of the criterion is the lowest among all the 15 candidate models.

Table 1, Table 2, Table 3, Table 4, Table 5, Table 6, Table 7, Table 8, Table 9, Table 10, Table 11 and Table 12 present the proportions of models selected by the considered criteria. Among the 15 candidate models only 4 were chosen at least once. These four models are the same in all instances and appear in the 2nd column of all tables.

For small sample sizes (n=20, n=30) the criteria PIC and MDIC yield the best results. When the level of contamination is 10% or 20%, the PIC criterion yields very good results and beats the other competitors almost all the time. When the level of contamination is small, for example 5% or when there is no contamination, the two criteria are comparable, in the sense that in many cases the proportions of selected models by the two criteria are very close, so that sometimes PIC wins and sometimes MDIC wins. Table 1, Table 2, Table 3 and Table 4 present these results for n=20, but similar results are obtained for n=30, too.

For medium sample sizes (n=50, n=75), the criteria PIC and BIC yield the best results. The results for n=50 are given in Table 5, Table 6, Table 7 and Table 8. Note that the PIC criterion yields the best results for 0% and 10% contamination. For the other levels of contamination, there are values of γ for which PIC is the best among all the considered criteria. On the other hand, in most cases when BIC wins, the proportions of selections of the true model by BIC and PIC are close.

When the sample size is large (n=100, n=200, n=500), BIC generally yields better results than PIC which stays relatively close behind, but sometimes BIC and PIC have the same performance. Table 9, Table 10, Table 11 and Table 12 present the results obtained for n=100.

Thus, the new PIC criterion works very well for small to medium sample sizes and for levels of contamination up to 20%, but falls behind BIC for large sample sizes. Note that for contaminated data, PIC with γ=0.15 prevails in most of the considered cases. On the other hand, for uncontaminated data, it is PIC with γ=0.2 that prevails in all the considered instances. It is also worth mentioning that PIC with γ=0.3 appears to behave very satisfactorily in most cases irrespectively of the proportion of contamination (0%–20%) and the sample size. Observe also that in all cases, AIC has the highest overestimation rate which is somehow expected (see [24]).

Although the consistency is the main focus of the applications presented in this work, one should point out that if prediction is part of the objective of a regression analysis, then model selection carried out using criteria such as the ones used in this work, have desirable properties. In fact, the case of finite-dimensional normal regression models has been shown to be associated with satisfactory prediction errors for criteria such as AIC and BIC (see [25]). Furthermore, it should be pointed out that in many instances PIC has a behavior quite similar to the above criteria by choosing the same models. Also, according to the presented simulation results, the proportion of choosing the true model by PIC is always better than the proportion of choosing the true model by AIC (even in the case of non contaminated data) and sometimes it is better than the proportion of choosing the true model by BIC. These results imply a satisfactory prediction ability for the proposed PIC criterion.

In conclusion, the new PIC criterion is a good competitor of the well known model selection criteria AIC, BIC and MDIC and may have superior performance especially in the case of small and contaminated samples.

### 4.2. Real Data Example

In order to illustrate the proposed method, we used the Hald cement data (see [26]) which represent a popular example for multiple linear regression. This example concern the heat evolved in calories per gram of cement *Y* as a function of the amount of each of four ingredient in the mix: tricalcium aluminate ($X_{1}$), tricalcium silicate ($X_{2}$), tetracalcium alumino-ferrite ($X_{3}$) and dicalcium silicate ($X_{4}$). The data are presented in Table 13.

Since 4 variables are available, there are 15 possible candidate models (involving at least one regressor) for this data set. Note that the 4 single-variable models should be excluded from the analysis, because cement involves a mixture of at least two components that react chemically (see [27], p. 102). The model selection criteria that have been used are PIC for several values of γ, AIC, BIC and MDIC with α=0.25. Table 14 shows the model selected by each of the considered criteria.

Observe that, in this example, PIC behaves similarly to AIC and MDIC having a slight tendency of overestimation. Note that for this specific dataset the collinearity is quite strong with $X_{1}$ and $X_{3}$ as well as $X_{2}$ and $X_{4}$ being seriously correlated. It should be pointed out that the model $\left(X_{1},X_{2},X_{4}\right)$ is chosen not only by AIC and PIC, but also by $C_{p}$ Mallows’ criterion ([1]) with $\left(X_{1},X_{2},X_{3}\right)$ coming very close second. Note further that $\left(X_{1},X_{2},X_{4}\right)$ has also been chosen by cross validation ([28], p. 33) and PRESS ([26], p. 325). Finally, it is worth noticing that these two models share the highest adjusted $R^{2}$ values which are almost identical (0.976 for $\left(X_{1},X_{2},X_{4}\right)$ and 0.974 for $\left(X_{1},X_{2},X_{3}\right)$) making the distinction between them extremely hard. Thus, in this example, the new PIC criterion gives results as good as other recognized classical model selection criteria.

## 5. Conclusions

In this work, by applying the same methodology as for AIC to a family of pseudodistances, we constructed new model selection criteria using minimum pseudodistance estimators. We proved theoretical properties of these criteria including asymptotic unbiasedness, robustness, consistency, as well as the limit laws. The case of the linear regression models was studied in detail and specific selection criteria based on pseudodistance are proposed.

For linear regression models, a comparative study based on Monte Carlo simulations illustrate the performance of the new methodology. Thus, for small sample sizes, the criteria PIC and MDIC yield the best results and in many cases PIC wins, for example when the level of contamination is 10% or 20%. For medium sample sizes, the criteria PIC and BIC yield the best results. When the sample size is large, BIC generally yields better results than PIC which stays relatively close behind, but sometimes BIC and PIC have the same performance.

Based on the results of the simulation study and on the real data example, we conclude that the new PIC criterion is a good competitor of the well known criteria AIC, BIC and MDIC with an overall performance which is very satisfactory for all possible settings according to the sample size and contamination rate. Also PIC may have superior performance, especially in the case of small and contaminated samples.

An important issue that needs further investigation is the choice of the appropriate value for the parameter γ associated to the procedure. The findings of the presented simulation study show that, for contaminated data, the value γ=0.15 leads to very good results, irrespectively of the sample size. Also, γ=0.3 produces overall very satisfactory results, irrespectively of the sample size and the contamination rate. We hope to explore further and provide a clear solution to this problem, in a future work. We also intend to extend this methodology to other type of models including nonlinear or time series models.

## Figures and Tables

**Table 1:** Proportions of selected models by the considered criteria (*n* = 20, $d_{1}=0.8$).

Criteria	Variables	γ=0.01	γ=0.05	γ=0.1	γ=0.15	γ=0.2	γ=0.25	γ=0.3
**PIC**	$X_{1},X_{2}$	**90**	84	**88**	**84**	**92**	**90**	**86**
	$X_{1},X_{2},X_{3}$	${\}}_{}^{\left(10\right)}$	${\}}_{}^{\left(16\right)}$	${\}}_{}^{\left(12\right)}$	${\}}_{}^{\left(16\right)}$	${\}}_{}^{\left(8\right)}$	${\}}_{}^{\left(10\right)}$	${\}}_{}^{\left(14\right)}$
	$X_{1},X_{2},X_{4}$							
	$X_{1},X_{2},X_{3},X_{4}$							
**AIC**	$X_{1},X_{2}$	62	56	52	56	66	56	60
	$X_{1},X_{2},X_{3}$	${\}}_{}^{\left(38\right)}$	${\}}_{}^{\left(44\right)}$	${\}}_{}^{\left(48\right)}$	${\}}_{}^{\left(44\right)}$	${\}}_{}^{\left(34\right)}$	${\}}_{}^{\left(44\right)}$	${\}}_{}^{\left(40\right)}$
	$X_{1},X_{2},X_{4}$							
	$X_{1},X_{2},X_{3},X_{4}$							
**BIC**	$X_{1},X_{2}$	74	76	60	74	72	68	70
	$X_{1},X_{2},X_{3}$	${\}}_{}^{\left(26\right)}$	${\}}_{}^{\left(24\right)}$	${\}}_{}^{\left(40\right)}$	${\}}_{}^{\left(26\right)}$	${\}}_{}^{\left(28\right)}$	${\}}_{}^{\left(32\right)}$	${\}}_{}^{\left(30\right)}$
	$X_{1},X_{2},X_{4}$							
	$X_{1},X_{2},X_{3},X_{4}$							
**MDIC**	$X_{1},X_{2}$	86	**86**	64	78	84	80	74
	$X_{1},X_{2},X_{3}$	${\}}_{}^{\left(14\right)}$	${\}}_{}^{\left(14\right)}$	${\}}_{}^{\left(36\right)}$	${\}}_{}^{\left(22\right)}$	${\}}_{}^{\left(16\right)}$	${\}}_{}^{\left(20\right)}$	${\}}_{}^{\left(26\right)}$
	$X_{1},X_{2},X_{4}$							
	$X_{1},X_{2},X_{3},X_{4}$							

**Table 2:** Proportions of selected models by the considered criteria (*n* = 20, $d_{1}=0.9$).

Criteria	Variables	γ=0.01	γ=0.05	γ=0.1	γ=0.15	γ=0.2	γ=0.25	γ=0.3
PIC	$X_{1},X_{2}$	80	**84**	**90**	**82**	82	**80**	80
	$X_{1},X_{2},X_{3}$	${\}}_{}^{\left(20\right)}$	${\}}_{}^{\left(16\right)}$	${\}}_{}^{\left(10\right)}$	${\}}_{}^{\left(18\right)}$	${\}}_{}^{\left(18\right)}$	${\}}_{}^{\left(20\right)}$	${\}}_{}^{\left(20\right)}$
	$X_{1},X_{2},X_{4}$							
	$X_{1},X_{2},X_{3},X_{4}$							
**AIC**	$X_{1},X_{2}$	60	52	56	62	64	54	52
	$X_{1},X_{2},X_{3}$	${\}}_{}^{\left(40\right)}$	${\}}_{}^{\left(48\right)}$	${\}}_{}^{\left(44\right)}$	${\}}_{}^{\left(38\right)}$	${\}}_{}^{\left(36\right)}$	${\}}_{}^{\left(46\right)}$	${\}}_{}^{\left(48\right)}$
	$X_{1},X_{2},X_{4}$							
	$X_{1},X_{2},X_{3},X_{4}$							
**BIC**	$X_{1},X_{2}$	76	70	78	72	84	76	76
	$X_{1},X_{2},X_{3}$	${\}}_{}^{\left(24\right)}$	${\}}_{}^{\left(30\right)}$	${\}}_{}^{\left(22\right)}$	${\}}_{}^{\left(28\right)}$	${\}}_{}^{\left(16\right)}$	${\}}_{}^{\left(24\right)}$	${\}}_{}^{\left(24\right)}$
	$X_{1},X_{2},X_{4}$							
	$X_{1},X_{2},X_{3},X_{4}$							
**MDIC**	$X_{1},X_{2}$	**86**	76	88	74	**92**	78	**86**
	$X_{1},X_{2},X_{3}$	${\}}_{}^{\left(14\right)}$	${\}}_{}^{\left(24\right)}$	${\}}_{}^{\left(22\right)}$	${\}}_{}^{\left(26\right)}$	${\}}_{}^{\left(8\right)}$	${\}}_{}^{\left(22\right)}$	${\}}_{}^{\left(14\right)}$
	$X_{1},X_{2},X_{4}$							
	$X_{1},X_{2},X_{3},X_{4}$							

**Table 3:** Proportions of selected models by the considered criteria (*n* = 20, $d_{1}=0.95$).

Criteria	Variables	γ=0.01	γ=0.05	γ=0.1	γ=0.15	γ=0.2	γ=0.25	γ=0.3
PIC	$X_{1},X_{2}$	82	**88**	80	**94**	82	**88**	86
	$X_{1},X_{2},X_{3}$	${\}}_{}^{\left(8\right)}$	${\}}_{}^{\left(12\right)}$	${\}}_{}^{\left(20\right)}$	${\}}_{}^{\left(6\right)}$	${\}}_{}^{\left(18\right)}$	${\}}_{}^{\left(12\right)}$	${\}}_{}^{\left(14\right)}$
	$X_{1},X_{2},X_{4}$							
	$X_{1},X_{2},X_{3},X_{4}$							
**AIC**	$X_{1},X_{2}$	78	50	66	70	66	64	66
	$X_{1},X_{2},X_{3}$	${\}}_{}^{\left(22\right)}$	${\}}_{}^{\left(50\right)}$	${\}}_{}^{\left(34\right)}$	${\}}_{}^{\left(30\right)}$	${\}}_{}^{\left(34\right)}$	${\}}_{}^{\left(36\right)}$	${\}}_{}^{\left(34\right)}$
	$X_{1},X_{2},X_{4}$							
	$X_{1},X_{2},X_{3},X_{4}$							
**BIC**	$X_{1},X_{2}$	84	64	74	84	84	76	82
	$X_{1},X_{2},X_{3}$	${\}}_{}^{\left(16\right)}$	${\}}_{}^{\left(36\right)}$	${\}}_{}^{\left(26\right)}$	${\}}_{}^{\left(16\right)}$	${\}}_{}^{\left(16\right)}$	${\}}_{}^{\left(24\right)}$	${\}}_{}^{\left(18\right)}$
	$X_{1},X_{2},X_{4}$							
	$X_{1},X_{2},X_{3},X_{4}$							
**MDIC**	$X_{1},X_{2}$	**90**	74	**82**	88	**88**	80	**88**
	$X_{1},X_{2},X_{3}$	${\}}_{}^{\left(10\right)}$	${\}}_{}^{\left(26\right)}$	${\}}_{}^{\left(18\right)}$	${\}}_{}^{\left(12\right)}$	${\}}_{}^{\left(12\right)}$	${\}}_{}^{\left(20\right)}$	${\}}_{}^{\left(12\right)}$
	$X_{1},X_{2},X_{4}$							
	$X_{1},X_{2},X_{3},X_{4}$							

**Table 4:** Proportions of selected models by the considered criteria (*n* = 20, $d_{1}=1$).

Criteria	Variables	γ=0.01	γ=0.05	γ=0.1	γ=0.15	γ=0.2	γ=0.25	γ=0.3
PIC	$X_{1},X_{2}$	**86**	86	86	86	**88**	82	**92**
	$X_{1},X_{2},X_{3}$	${\}}_{}^{\left(14\right)}$	${\}}_{}^{\left(14\right)}$	${\}}_{}^{\left(14\right)}$	${\}}_{}^{\left(14\right)}$	${\}}_{}^{\left(12\right)}$	${\}}_{}^{\left(18\right)}$	${\}}_{}^{\left(8\right)}$
	$X_{1},X_{2},X_{4}$							
	$X_{1},X_{2},X_{3},X_{4}$							
**AIC**	$X_{1},X_{2}$	64	74	62	58	64	62	70
	$X_{1},X_{2},X_{3}$	${\}}_{}^{\left(36\right)}$	${\}}_{}^{\left(26\right)}$	${\}}_{}^{\left(38\right)}$	${\}}_{}^{\left(42\right)}$	${\}}_{}^{\left(36\right)}$	${\}}_{}^{\left(38\right)}$	${\}}_{}^{\left(30\right)}$
	$X_{1},X_{2},X_{4}$							
	$X_{1},X_{2},X_{3},X_{4}$							
**BIC**	$X_{1},X_{2}$	78	90	78	80	82	80	74
	$X_{1},X_{2},X_{3}$	${\}}_{}^{\left(22\right)}$	${\}}_{}^{\left(10\right)}$	${\}}_{}^{\left(22\right)}$	${\}}_{}^{\left(20\right)}$	${\}}_{}^{\left(18\right)}$	${\}}_{}^{\left(20\right)}$	${\}}_{}^{\left(26\right)}$
	$X_{1},X_{2},X_{4}$							
	$X_{1},X_{2},X_{3},X_{4}$							
**MDIC**	$X_{1},X_{2}$	84	**92**	**88**	**88**	**88**	**88**	80
	$X_{1},X_{2},X_{3}$	${\}}_{}^{\left(16\right)}$	${\}}_{}^{\left(8\right)}$	${\}}_{}^{\left(12\right)}$	${\}}_{}^{\left(12\right)}$	${\}}_{}^{\left(12\right)}$	${\}}_{}^{\left(12\right)}$	${\}}_{}^{\left(20\right)}$
	$X_{1},X_{2},X_{4}$							
	$X_{1},X_{2},X_{3},X_{4}$							

**Table 5:** Proportions of selected models by the considered criteria (*n* = 50, $d_{1}=0.8$).

Criteria	Variables	γ=0.01	γ=0.05	γ=0.1	γ=0.15	γ=0.2	γ=0.25	γ=0.3
PIC	$X_{1},X_{2}$	86	**96**	94	**90**	88	86	**90**
	$X_{1},X_{2},X_{3}$	${\}}_{}^{\left(14\right)}$	${\}}_{}^{\left(4\right)}$	${\}}_{}^{\left(6\right)}$	${\}}_{}^{\left(10\right)}$	${\}}_{}^{\left(12\right)}$	${\}}_{}^{\left(14\right)}$	${\}}_{}^{\left(10\right)}$
	$X_{1},X_{2},X_{4}$							
	$X_{1},X_{2},X_{3},X_{4}$							
**AIC**	$X_{1},X_{2}$	74	64	82	62	64	78	72
	$X_{1},X_{2},X_{3}$	${\}}_{}^{\left(26\right)}$	${\}}_{}^{\left(36\right)}$	${\}}_{}^{\left(18\right)}$	${\}}_{}^{\left(38\right)}$	${\}}_{}^{\left(36\right)}$	${\}}_{}^{\left(22\right)}$	${\}}_{}^{\left(28\right)}$
	$X_{1},X_{2},X_{4}$							
	$X_{1},X_{2},X_{3},X_{4}$							
**BIC**	$X_{1},X_{2}$	**94**	86	**96**	86	**90**	**88**	**90**
	$X_{1},X_{2},X_{3}$	${\}}_{}^{\left(6\right)}$	${\}}_{}^{\left(14\right)}$	${\}}_{}^{\left(4\right)}$	${\}}_{}^{\left(14\right)}$	${\}}_{}^{\left(10\right)}$	${\}}_{}^{\left(12\right)}$	${\}}_{}^{\left(10\right)}$
	$X_{1},X_{2},X_{4}$							
	$X_{1},X_{2},X_{3},X_{4}$							
**MDIC**	$X_{1},X_{2}$	**94**	82	98	82	86	**88**	**90**
	$X_{1},X_{2},X_{3}$	${\}}_{}^{\left(6\right)}$	${\}}_{}^{\left(18\right)}$	${\}}_{}^{\left(2\right)}$	${\}}_{}^{\left(18\right)}$	${\}}_{}^{\left(14\right)}$	${\}}_{}^{\left(12\right)}$	${\}}_{}^{\left(10\right)}$
	$X_{1},X_{2},X_{4}$							
	$X_{1},X_{2},X_{3},X_{4}$							

**Table 6:** Proportions of selected models by the considered criteria (*n* = 50, $d_{1}=0.9$).

Criteria	Variables	γ=0.01	γ=0.05	γ=0.1	γ=0.15	γ=0.2	γ=0.25	γ=0.3
PIC	$X_{1},X_{2}$	**92**	**88**	**92**	90	82	**94**	**86**
	$X_{1},X_{2},X_{3}$	${\}}_{}^{\left(8\right)}$	${\}}_{}^{\left(12\right)}$	${\}}_{}^{\left(8\right)}$	${\}}_{}^{\left(10\right)}$	${\}}_{}^{\left(18\right)}$	${\}}_{}^{\left(6\right)}$	${\}}_{}^{\left(14\right)}$
	$X_{1},X_{2},X_{4}$							
	$X_{1},X_{2},X_{3},X_{4}$							
**AIC**	$X_{1},X_{2}$	70	64	62	64	66	74	72
	$X_{1},X_{2},X_{3}$	${\}}_{}^{\left(30\right)}$	${\}}_{}^{\left(36\right)}$	${\}}_{}^{\left(38\right)}$	${\}}_{}^{\left(36\right)}$	${\}}_{}^{\left(34\right)}$	${\}}_{}^{\left(26\right)}$	${\}}_{}^{\left(28\right)}$
	$X_{1},X_{2},X_{4}$							
	$X_{1},X_{2},X_{3},X_{4}$							
**BIC**	$X_{1},X_{2}$	**92**	**88**	82	**92**	**88**	88	**86**
	$X_{1},X_{2},X_{3}$	${\}}_{}^{\left(8\right)}$	${\}}_{}^{\left(12\right)}$	${\}}_{}^{\left(18\right)}$	${\}}_{}^{\left(8\right)}$	${\}}_{}^{\left(12\right)}$	${\}}_{}^{\left(12\right)}$	${\}}_{}^{\left(14\right)}$
	$X_{1},X_{2},X_{4}$							
	$X_{1},X_{2},X_{3},X_{4}$							
**MDIC**	$X_{1},X_{2}$	**92**	86	76	88	84	88	**86**
	$X_{1},X_{2},X_{3}$	${\}}_{}^{\left(8\right)}$	${\}}_{}^{\left(14\right)}$	${\}}_{}^{\left(24\right)}$	${\}}_{}^{\left(12\right)}$	${\}}_{}^{\left(16\right)}$	${\}}_{}^{\left(12\right)}$	${\}}_{}^{\left(14\right)}$
	$X_{1},X_{2},X_{4}$							
	$X_{1},X_{2},X_{3},X_{4}$							

**Table 7:** Proportions of selected models by the considered criteria (*n* = 50, $d_{1}=0.95$).

Criteria	Variables	γ=0.01	γ=0.05	γ=0.1	γ=0.15	γ=0.2	γ=0.25	γ=0.3
PIC	$X_{1},X_{2}$	94	**92**	**92**	**88**	84	90	**88**
	$X_{1},X_{2},X_{3}$	${\}}_{}^{\left(6\right)}$	${\}}_{}^{\left(8\right)}$	${\}}_{}^{\left(8\right)}$	${\}}_{}^{\left(12\right)}$	${\}}_{}^{\left(16\right)}$	${\}}_{}^{\left(10\right)}$	${\}}_{}^{\left(12\right)}$
	$X_{1},X_{2},X_{4}$							
	$X_{1},X_{2},X_{3},X_{4}$							
**AIC**	$X_{1},X_{2}$	70	62	66	68	70	72	58
	$X_{1},X_{2},X_{3}$	${\}}_{}^{\left(30\right)}$	${\}}_{}^{\left(38\right)}$	${\}}_{}^{\left(34\right)}$	${\}}_{}^{\left(32\right)}$	${\}}_{}^{\left(30\right)}$	${\}}_{}^{\left(28\right)}$	${\}}_{}^{\left(42\right)}$
	$X_{1},X_{2},X_{4}$							
	$X_{1},X_{2},X_{3},X_{4}$							
**BIC**	$X_{1},X_{2}$	**96**	82	**92**	86	**92**	**92**	86
	$X_{1},X_{2},X_{3}$	${\}}_{}^{\left(4\right)}$	${\}}_{}^{\left(18\right)}$	${\}}_{}^{\left(8\right)}$	${\}}_{}^{\left(14\right)}$	${\}}_{}^{\left(8\right)}$	${\}}_{}^{\left(8\right)}$	${\}}_{}^{\left(14\right)}$
	$X_{1},X_{2},X_{4}$							
	$X_{1},X_{2},X_{3},X_{4}$							
**MDIC**	$X_{1},X_{2}$	90	78	88	86	86	90	82
	$X_{1},X_{2},X_{3}$	${\}}_{}^{\left(10\right)}$	${\}}_{}^{\left(22\right)}$	${\}}_{}^{\left(12\right)}$	${\}}_{}^{\left(14\right)}$	${\}}_{}^{\left(14\right)}$	${\}}_{}^{\left(10\right)}$	${\}}_{}^{\left(18\right)}$
	$X_{1},X_{2},X_{4}$							
	$X_{1},X_{2},X_{3},X_{4}$							

**Table 8:** Proportions of selected models by the considered criteria (*n* = 50, $d_{1}=1$).

Criteria	Variables	γ=0.01	γ=0.05	γ=0.1	γ=0.15	γ=0.2	γ=0.25	γ=0.3
PIC	$X_{1},X_{2}$	**94**	**90**	80	84	**90**	**94**	**88**
	$X_{1},X_{2},X_{3}$	${\}}_{}^{\left(6\right)}$	${\}}_{}^{\left(10\right)}$	${\}}_{}^{\left(20\right)}$	${\}}_{}^{\left(16\right)}$	${\}}_{}^{\left(10\right)}$	${\}}_{}^{\left(6\right)}$	${\}}_{}^{\left(12\right)}$
	$X_{1},X_{2},X_{4}$							
	$X_{1},X_{2},X_{3},X_{4}$							
**AIC**	$X_{1},X_{2}$	64	68	62	68	66	64	62
	$X_{1},X_{2},X_{3}$	${\}}_{}^{\left(34\right)}$	${\}}_{}^{\left(32\right)}$	${\}}_{}^{\left(38\right)}$	${\}}_{}^{\left(32\right)}$	${\}}_{}^{\left(34\right)}$	${\}}_{}^{\left(36\right)}$	${\}}_{}^{\left(38\right)}$
	$X_{1},X_{2},X_{4}$							
	$X_{1},X_{2},X_{3},X_{4}$							
**BIC**	$X_{1},X_{2}$	86	86	**86**	**90**	86	**94**	82
	$X_{1},X_{2},X_{3}$	${\}}_{}^{\left(14\right)}$	${\}}_{}^{\left(14\right)}$	${\}}_{}^{\left(14\right)}$	${\}}_{}^{\left(10\right)}$	${\}}_{}^{\left(14\right)}$	${\}}_{}^{\left(6\right)}$	${\}}_{}^{\left(18\right)}$
	$X_{1},X_{2},X_{4}$							
	$X_{1},X_{2},X_{3},X_{4}$							
**MDIC**	$X_{1},X_{2}$	84	84	82	88	84	90	82
	$X_{1},X_{2},X_{3}$	${\}}_{}^{\left(16\right)}$	${\}}_{}^{\left(16\right)}$	${\}}_{}^{\left(18\right)}$	${\}}_{}^{\left(12\right)}$	${\}}_{}^{\left(16\right)}$	${\}}_{}^{\left(10\right)}$	${\}}_{}^{\left(18\right)}$
	$X_{1},X_{2},X_{4}$							
	$X_{1},X_{2},X_{3},X_{4}$							

**Table 9:** Proportions of selected models by the considered criteria (*n* = 100, $d_{1}=0.8$).

Criteria	Variables	γ=0.01	γ=0.05	γ=0.1	γ=0.15	γ=0.2	γ=0.25	γ=0.3
PIC	$X_{1},X_{2}$	**94**	94	94	**92**	88	88	**94**
	$X_{1},X_{2},X_{3}$	${\}}_{}^{\left(6\right)}$	${\}}_{}^{\left(6\right)}$	${\}}_{}^{\left(6\right)}$	${\}}_{}^{\left(8\right)}$	${\}}_{}^{\left(12\right)}$	${\}}_{}^{\left(12\right)}$	${\}}_{}^{\left(6\right)}$
	$X_{1},X_{2},X_{4}$							
	$X_{1},X_{2},X_{3},X_{4}$							
**AIC**	$X_{1},X_{2}$	70	82	78	70	68	68	72
	$X_{1},X_{2},X_{3}$	${\}}_{}^{\left(30\right)}$	${\}}_{}^{\left(18\right)}$	${\}}_{}^{\left(22\right)}$	${\}}_{}^{\left(30\right)}$	${\}}_{}^{\left(32\right)}$	${\}}_{}^{\left(32\right)}$	${\}}_{}^{\left(28\right)}$
	$X_{1},X_{2},X_{4}$							
	$X_{1},X_{2},X_{3},X_{4}$							
**BIC**	$X_{1},X_{2}$	90	**96**	**98**	90	**96**	**94**	88
	$X_{1},X_{2},X_{3}$	${\}}_{}^{\left(10\right)}$	${\}}_{}^{\left(4\right)}$	${\}}_{}^{\left(2\right)}$	${\}}_{}^{\left(10\right)}$	${\}}_{}^{\left(4\right)}$	${\}}_{}^{\left(6\right)}$	${\}}_{}^{\left(12\right)}$
	$X_{1},X_{2},X_{4}$							
	$X_{1},X_{2},X_{3},X_{4}$							
**MDIC**	$X_{1},X_{2}$	86	96	92	86	92	90	88
	$X_{1},X_{2},X_{3}$	${\}}_{}^{\left(14\right)}$	${\}}_{}^{\left(4\right)}$	${\}}_{}^{\left(8\right)}$	${\}}_{}^{\left(14\right)}$	${\}}_{}^{\left(8\right)}$	${\}}_{}^{\left(10\right)}$	${\}}_{}^{\left(12\right)}$
	$X_{1},X_{2},X_{4}$							
	$X_{1},X_{2},X_{3},X_{4}$							

**Table 10:** Proportions of selected models by the considered criteria (*n* = 100, $d_{1}=0.9$).

Criteria	Variables	γ=0.01	γ=0.05	γ=0.1	γ=0.15	γ=0.2	γ=0.25	γ=0.3
PIC	$X_{1},X_{2}$	88	92	**96**	**88**	88	88	86
	$X_{1},X_{2},X_{3}$	${\}}_{}^{\left(12\right)}$	${\}}_{}^{\left(8\right)}$	${\}}_{}^{\left(4\right)}$	${\}}_{}^{\left(12\right)}$	${\}}_{}^{\left(12\right)}$	${\}}_{}^{\left(12\right)}$	${\}}_{}^{\left(14\right)}$
	$X_{1},X_{2},X_{4}$							
	$X_{1},X_{2},X_{3},X_{4}$							
**AIC**	$X_{1},X_{2}$	68	72	78	66	70	78	60
	$X_{1},X_{2},X_{3}$	${\}}_{}^{\left(32\right)}$	${\}}_{}^{\left(28\right)}$	${\}}_{}^{\left(22\right)}$	${\}}_{}^{\left(34\right)}$	${\}}_{}^{\left(30\right)}$	${\}}_{}^{\left(22\right)}$	${\}}_{}^{\left(40\right)}$
	$X_{1},X_{2},X_{4}$							
	$X_{1},X_{2},X_{3},X_{4}$							
**BIC**	$X_{1},X_{2}$	**98**	**98**	**96**	**88**	**92**	**94**	**92**
	$X_{1},X_{2},X_{3}$	${\}}_{}^{\left(2\right)}$	${\}}_{}^{\left(2\right)}$	${\}}_{}^{\left(4\right)}$	${\}}_{}^{\left(12\right)}$	${\}}_{}^{\left(8\right)}$	${\}}_{}^{\left(6\right)}$	${\}}_{}^{\left(8\right)}$
	$X_{1},X_{2},X_{4}$							
	$X_{1},X_{2},X_{3},X_{4}$							
**MDIC**	$X_{1},X_{2}$	90	90	**96**	84	82	90	82
	$X_{1},X_{2},X_{3}$	${\}}_{}^{\left(10\right)}$	${\}}_{}^{\left(10\right)}$	${\}}_{}^{\left(4\right)}$	${\}}_{}^{\left(16\right)}$	${\}}_{}^{\left(18\right)}$	${\}}_{}^{\left(10\right)}$	${\}}_{}^{\left(18\right)}$
	$X_{1},X_{2},X_{4}$							
	$X_{1},X_{2},X_{3},X_{4}$							

**Table 11:** Proportions of selected models by the considered criteria (*n* = 100, $d_{1}=0.95$).

Criteria	Variables	γ=0.01	γ=0.05	γ=0.1	γ=0.15	γ=0.2	γ=0.25	γ=0.3
PIC	$X_{1},X_{2}$	90	88	**92**	90	**98**	**96**	**92**
	$X_{1},X_{2},X_{3}$	${\}}_{}^{\left(10\right)}$	${\}}_{}^{\left(12\right)}$	${\}}_{}^{\left(8\right)}$	${\}}_{}^{\left(10\right)}$	${\}}_{}^{\left(2\right)}$	${\}}_{}^{\left(4\right)}$	${\}}_{}^{\left(8\right)}$
	$X_{1},X_{2},X_{4}$							
	$X_{1},X_{2},X_{3},X_{4}$							
**AIC**	$X_{1},X_{2}$	70	78	78	66	82	68	68
	$X_{1},X_{2},X_{3}$	${\}}_{}^{\left(30\right)}$	${\}}_{}^{\left(22\right)}$	${\}}_{}^{\left(22\right)}$	${\}}_{}^{\left(34\right)}$	${\}}_{}^{\left(18\right)}$	${\}}_{}^{\left(32\right)}$	${\}}_{}^{\left(32\right)}$
	$X_{1},X_{2},X_{4}$							
	$X_{1},X_{2},X_{3},X_{4}$							
**BIC**	$X_{1},X_{2}$	**96**	**92**	**92**	**94**	96	94	88
	$X_{1},X_{2},X_{3}$	${\}}_{}^{\left(4\right)}$	${\}}_{}^{\left(8\right)}$	${\}}_{}^{\left(8\right)}$	${\}}_{}^{\left(6\right)}$	${\}}_{}^{\left(4\right)}$	${\}}_{}^{\left(6\right)}$	${\}}_{}^{\left(12\right)}$
	$X_{1},X_{2},X_{4}$							
	$X_{1},X_{2},X_{3},X_{4}$							
**MDIC**	$X_{1},X_{2}$	90	88	82	90	94	84	88
	$X_{1},X_{2},X_{3}$	${\}}_{}^{\left(10\right)}$	${\}}_{}^{\left(12\right)}$	${\}}_{}^{\left(18\right)}$	${\}}_{}^{\left(10\right)}$	${\}}_{}^{\left(6\right)}$	${\}}_{}^{\left(16\right)}$	${\}}_{}^{\left(12\right)}$
	$X_{1},X_{2},X_{4}$							
	$X_{1},X_{2},X_{3},X_{4}$							

**Table 12:** Proportions of the selected models by the considered criteria (*n* = 100, $d_{1}=1$).

Criteria	Variables	γ=0.01	γ=0.05	γ=0.1	γ=0.15	γ=0.2	γ=0.25	γ=0.3
PIC	$X_{1},X_{2}$	94	96	**92**	92	**96**	**90**	94
	$X_{1},X_{2},X_{3}$	${\}}_{}^{\left(6\right)}$	${\}}_{}^{\left(4\right)}$	${\}}_{}^{\left(8\right)}$	${\}}_{}^{\left(8\right)}$	${\}}_{}^{\left(4\right)}$	${\}}_{}^{\left(10\right)}$	${\}}_{}^{\left(6\right)}$
	$X_{1},X_{2},X_{4}$							
	$X_{1},X_{2},X_{3},X_{4}$							
**AIC**	$X_{1},X_{2}$	78	74	72	74	70	62	74
	$X_{1},X_{2},X_{3}$	${\}}_{}^{\left(22\right)}$	${\}}_{}^{\left(26\right)}$	${\}}_{}^{\left(28\right)}$	${\}}_{}^{\left(26\right)}$	${\}}_{}^{\left(30\right)}$	${\}}_{}^{\left(38\right)}$	${\}}_{}^{\left(26\right)}$
	$X_{1},X_{2},X_{4}$							
	$X_{1},X_{2},X_{3},X_{4}$							
**BIC**	$X_{1},X_{2}$	**96**	**100**	**92**	**96**	94	**90**	**100**
	$X_{1},X_{2},X_{3}$	${\}}_{}^{\left(4\right)}$		${\}}_{}^{\left(8\right)}$	${\}}_{}^{\left(4\right)}$	${\}}_{}^{\left(6\right)}$	${\}}_{}^{\left(10\right)}$	
	$X_{1},X_{2},X_{4}$							
	$X_{1},X_{2},X_{3},X_{4}$							
**MDIC**	$X_{1},X_{2}$	94	92	86	90	86	80	94
	$X_{1},X_{2},X_{3}$	${\}}_{}^{\left(6\right)}$	${\}}_{}^{\left(8\right)}$	${\}}_{}^{\left(14\right)}$	${\}}_{}^{\left(10\right)}$	${\}}_{}^{\left(14\right)}$	${\}}_{}^{\left(20\right)}$	${\}}_{}^{\left(6\right)}$
	$X_{1},X_{2},X_{4}$							
	$X_{1},X_{2},X_{3},X_{4}$							

**Table 13:** Hald cement data.

$X_{1}$	$X_{2}$	$X_{3}$	$X_{4}$	*Y*
7	26	6	60	78.5
1	29	15	52	74.3
11	56	8	20	104.3
11	31	8	47	87.6
7	52	6	33	95.9
11	55	9	22	109.2
3	71	17	6	102.7
1	31	22	44	72.5
2	54	18	22	93.1
21	47	4	26	115.9
1	40	23	34	83.8
11	66	9	12	113.3
10	68	8	12	109.4

**Table 14:** Selected models by model selection criteria.

Criteria	Variables
**PIC**, γ=0.05	$X_{1},X_{2},X_{4}$
**PIC**, γ=0.15	$X_{1},X_{2},X_{4}$
**PIC**, γ=0.2	$X_{1},X_{2},X_{3}$
**PIC**, γ=0.25	$X_{1},X_{2},X_{4}$
**PIC**, γ=0.3	$X_{1},X_{2},X_{4}$
**AIC**	$X_{1},X_{2},X_{4}$
**BIC**	$X_{1},X_{2}$
**MDIC**	$X_{1},X_{2},X_{3}$

## Abbreviations

The following abbreviations are used in this manuscript:

AIC	Akaike Information Criterion
BIC	Bayesian Information Criterion
GIC	General Information Criterion
DIC	Divergence Information Criterion
MDIC	Modified Divergence Information Criterion
PIC	Pseudodistance based Information Criterion
BHHJ family of measures	Basu, Harris, Hjort and Jones family of measures

## Appendix A

**Proof** **of Proposition 1.** Using a Taylor expansion of $W_{\theta }$ around the true parameter $\theta_{0}$ and taking $\theta ={\hat{\theta }}_{n}$, on the basis of (12) and (13) we obtain

(A1) $$W_{{\hat{\theta }}_{n}}=W_{\theta_{0}}+\frac{1}{2}{\left({\hat{\theta }}_{n}-\theta_{0}\right)}^{t}M_{\gamma }\left(\theta_{0}\right)\left({\hat{\theta }}_{n}-\theta_{0}\right)+o(∥{\hat{\theta }}_{n}-\theta_{0}{∥}^{2}).$$

Then (15) holds. □

**Proof** **of Proposition 2.** Using a Taylor expansion of $Q_{\theta }$ around to ${\hat{\theta }}_{n}$ and taking $\theta =\theta_{0}$, we obtain

(A2) $$Q_{\theta_{0}}=Q_{{\hat{\theta }}_{n}}+{\left[\frac{\partial }{\partial \theta }Q_{\theta }\right]}_{\theta ={\hat{\theta }}_{n}}^{t}\left(\theta_{0}-{\hat{\theta }}_{n}\right)+\frac{1}{2}{\left(\theta_{0}-{\hat{\theta }}_{n}\right)}^{t}{\left[\frac{\partial^{2}}{\partial \theta^{2}}Q_{\theta }\right]}_{\theta ={\hat{\theta }}_{n}}\left(\theta_{0}-{\hat{\theta }}_{n}\right)+o(∥{\hat{\theta }}_{n}-\theta_{0}{∥}^{2}).$$

Note that ${\left[\frac{\partial }{\partial \theta }Q_{\theta }\right]}_{\theta ={\hat{\theta }}_{n}}=0$ by the very definition of ${\hat{\theta }}_{n}$. By applying the weak law of large numbers and the continuous mapping theorem, we get

(A3) $${\left[\frac{\partial^{2}}{\partial \theta^{2}}Q_{\theta }\right]}_{\theta =\theta_{0}}-{\left[\frac{\partial^{2}}{\partial \theta^{2}}W_{\theta }\right]}_{\theta =\theta_{0}}\overset{P}{\to }0$$

and using (13)

(A4) $${\left[\frac{\partial^{2}}{\partial \theta^{2}}Q_{\theta }\right]}_{\theta =\theta_{0}}-M_{\gamma }\left(\theta_{0}\right)\overset{P}{\to }0.$$

Then, using the consistency of ${\hat{\theta }}_{n}$ and (A4), we obtain

(A5) $${\left[\frac{\partial^{2}}{\partial \theta^{2}}Q_{\theta }\right]}_{\theta ={\hat{\theta }}_{n}}=M_{\gamma }\left(\theta_{0}\right)+o_{P}\left(1\right).$$

Consequently,

(A6) $$Q_{\theta_{0}}=Q_{{\hat{\theta }}_{n}}+\frac{1}{2}{\left(\theta_{0}-{\hat{\theta }}_{n}\right)}^{t}M_{\gamma }\left(\theta_{0}\right)\left(\theta_{0}-{\hat{\theta }}_{n}\right)+o(∥{\hat{\theta }}_{n}-\theta_{0}{∥}^{2})$$

and we deduce (17). □

**Proof** **of Proposition 3.** Using Proposition 1 and Proposition 2, we obtain

(A7) $$E\left[W_{{\hat{\theta }}_{n}}\right]=E\left[Q_{{\hat{\theta }}_{n}}\right]+E\left[{\left(\theta_{0}-{\hat{\theta }}_{n}\right)}^{t}M_{\gamma }\left(\theta_{0}\right)\left(\theta_{0}-{\hat{\theta }}_{n}\right)\right]-E\left[Q_{\theta_{0}}\right]+W_{\theta_{0}}+E\left[R_{n}\right]$$

where $R_{n}=o(∥{\hat{\theta }}_{n}-\theta_{0}{∥}^{2})$. In order to evaluate $W_{\theta_{0}}-E\left[Q_{\theta_{0}}\right]$, note that

(A8) $$Q_{\theta_{0}}-W_{\theta_{0}}=-\frac{1}{\gamma }\left[\ln\left(\frac{1}{n}\sum_{i=1}^{n}p_{\theta_{0}}^{\gamma }\left(X_{i}\right)\right)-\ln\left(\int p_{\theta_{0}}^{\gamma +1}d\lambda \right)\right].$$

A Taylor expansion of the function lnx around to $\int p_{\theta_{0}}^{\gamma +1}d\lambda$ yields

(A9) $$\begin{array}{ccc} \ln\left(\frac{1}{n}\sum_{i=1}^{n}p_{\theta_{0}}^{\gamma }\left(X_{i}\right)\right) & = & \ln\left(\int p_{\theta_{0}}^{\gamma +1}d\lambda \right)+\frac{1}{\int p_{\theta_{0}}^{\gamma +1}d\lambda }\left[\frac{1}{n}\sum_{i=1}^{n}p_{\theta_{0}}^{\gamma }\left(X_{i}\right)-\int p_{\theta_{0}}^{\gamma +1}d\lambda \right]-\\ & & -\frac{1}{2}\cdot \frac{1}{{\left(\int p_{\theta_{0}}^{\gamma +1}d\lambda \right)}^{2}}{\left[\frac{1}{n}\sum_{i=1}^{n}p_{\theta_{0}}^{\gamma }\left(X_{i}\right)-\int p_{\theta_{0}}^{\gamma +1}d\lambda \right]}^{2}+\\ & & +o(∥\frac{1}{n}\sum_{i=1}^{n}p_{\theta_{0}}^{\gamma }\left(X_{i}\right)-\int p_{\theta_{0}}^{\gamma +1}d\lambda {∥}^{2}). \end{array}$$

Then

$$\begin{array}{ccc} E[Q_{\theta_{0}}-W_{\theta_{0}}] & = & -\frac{1}{\gamma }E\left[\ln\left(\frac{1}{n}\sum_{i=1}^{n}p_{\theta_{0}}^{\gamma }\left(X_{i}\right)\right)-\ln\left(\int p_{\theta_{0}}^{\gamma +1}d\lambda \right)\right]\\ & = & -\frac{1}{\gamma }\left\{\frac{1}{\int p_{\theta_{0}}^{\gamma +1}d\lambda }E\left[\frac{1}{n}\sum_{i=1}^{n}p_{\theta_{0}}^{\gamma }\left(X_{i}\right)-\int p_{\theta_{0}}^{\gamma +1}d\lambda \right]-\right.\\ & & \left.-\frac{1}{2}\cdot \frac{1}{{\left(\int p_{\theta_{0}}^{\gamma +1}d\lambda \right)}^{2}}E\left[{\left(\frac{1}{n}\sum_{i=1}^{n}p_{\theta_{0}}^{\gamma }\left(X_{i}\right)-\int p_{\theta_{0}}^{\gamma +1}d\lambda \right)}^{2}\right]+E\left[R_{n}^{\prime }\right]\right\} \end{array}$$

where $R_{n}^{\prime }=o(∥\frac{1}{n}\sum_{i=1}^{n}p_{\theta_{0}}^{\gamma }\left(X_{i}\right)-\int p_{\theta_{0}}^{\gamma +1}d\lambda {∥}^{2})$. On the other hand,

(A10) $$\begin{array}{ccc} E\left[{\left(\frac{1}{n}\sum_{i=1}^{n}p_{\theta_{0}}^{\gamma }\left(X_{i}\right)-\int p_{\theta_{0}}^{\gamma +1}d\lambda \right)}^{2}\right] & = & \mathrm{Var}\left[\frac{1}{n}\sum_{i=1}^{n}p_{\theta_{0}}^{\gamma }\left(X_{i}\right)\right]=\frac{1}{n}\mathrm{Var}\left[p_{\theta_{0}}^{\gamma }\left(X\right)\right]\\ & = & \frac{1}{n}\left\{E\left[p_{\theta_{0}}^{2\gamma }\left(X\right)\right]-E{\left[p_{\theta_{0}}^{\gamma }\left(X\right)\right]}^{2}\right\}\\ & = & \frac{\int p_{\theta_{0}}^{2\gamma +1}d\lambda -{\left(\int p_{\theta_{0}}^{\gamma +1}d\lambda \right)}^{2}}{n}. \end{array}$$

Consequently,

(A11) $$E\left[Q_{\theta_{0}}\right]-W_{\theta_{0}}=-\frac{1}{2\gamma n}\left[1-\frac{\int p_{\theta_{0}}^{2\gamma +1}d\lambda }{{\left(\int p_{\theta_{0}}^{\gamma +1}d\lambda \right)}^{2}}\right]-\frac{1}{\gamma }E\left[R_{n}^{\prime }\right].$$

Using (A7) and (A11), we obtain (18). □

**Proof** **of Proposition 5.** For the contaminated model ${\tilde{P}}_{\varepsilon x}=\left(1-\varepsilon \right)P_{\theta_{0}}+\varepsilon \delta_{x}$, it holds

(A12) $$U\left({\tilde{P}}_{\varepsilon x}\right)=\frac{1}{\gamma +1}\ln\left(\int p_{T({\tilde{P}}_{\varepsilon x})}^{\gamma +1}d\lambda \right)-\frac{1}{\gamma }\ln\left(\int p_{T({\tilde{P}}_{\varepsilon x})}^{\gamma }d{\tilde{P}}_{\varepsilon x}\right).$$

Derivation with respect to ε yields

$$\begin{array}{ccc} \frac{\partial }{\partial \varepsilon }{\left[U\left({\tilde{P}}_{\varepsilon x}\right)\right]}_{\varepsilon =0} & = & \frac{1}{\int p_{\theta_{0}}^{\gamma +1}d\lambda }\cdot \int p_{\theta_{0}}^{\gamma }{\dot{p}}_{\theta_{0}}d\lambda \cdot \mathrm{IF}\left(x;T,P_{\theta_{0}}\right)-\\ & & -\frac{1}{\gamma }\cdot \frac{1}{\int p_{\theta_{0}}^{\gamma +1}d\lambda }\left\{-\int p_{\theta_{0}}^{\gamma +1}d\lambda +\gamma \cdot \int p_{\theta_{0}}^{\gamma }{\dot{p}}_{\theta_{0}}d\lambda \cdot \mathrm{IF}\left(x;T,P_{\theta_{0}}\right)+p_{\theta_{0}}^{\gamma }\left(x\right)\right\}\\ & = & \frac{1}{\gamma }\cdot \left[1-\frac{p_{\theta_{0}}^{\gamma }\left(x\right)}{\int p_{\theta_{0}}^{\gamma +1}d\lambda }\right]. \end{array}$$

Thus we obtain

(A13) $$\mathrm{IF}\left(x;U,P_{\theta_{0}}\right)=\frac{1}{\gamma }\left[1-\frac{p_{\theta_{0}}^{\gamma }\left(x\right)}{\int p_{\theta_{0}}^{\gamma +1}d\lambda }\right].$$

□
